# High-Entropy Materials: A New Paradigm in the Design of Advanced Batteries

**DOI:** 10.1007/s40820-025-01842-w

**Published:** 2025-07-17

**Authors:** Yangmei Xin, Minmin Zhu, Haizhong Zhang, Xinghui Wang

**Affiliations:** 1 College of Physics and Information Engineering, Fuzhou University, Fuzhou, 350108 People’s Republic of China; 2 FZU-Jinjiang Joint Institute of Microelectronics, Jinjiang Science and Education Park, Fuzhou University, Jinjiang, 362200 People’s Republic of China

**Keywords:** High entropy alloys, High entropy oxides, High entropy MXenes, High entropy battery materials, Machine learning

## Abstract

High-entropy materials (HEMs) have attracted considerable research attention in battery applications due to exceptional properties such as remarkable structural stability, enhanced ionic conductivity, superior mechanical strength, and outstanding catalytic activity. These distinctive characteristics render HEMs highly suitable for various battery components, such as electrodes, electrolytes, and catalysts. This review systematically examines recent advances in the application of HEMs for energy storage, beginning with fundamental concepts, historical development, and key definitions. Three principal categories of HEMs, namely high-entropy alloys, high-entropy oxides, and high-entropy MXenes, are analyzed with a focus on electrochemical performance metrics such as specific capacity, energy density, cycling stability, and rate capability. The underlying mechanisms by which these materials enhance battery performance are elucidated in the discussion. Furthermore, the pivotal role of machine learning in accelerating the discovery and optimization of novel high-entropy battery materials is highlighted. The review concludes by outlining future research directions and potential breakthroughs in HEM-based battery technologies.

## Highlights

- The development history, characteristics and applications of high entropy alloys, high entropy oxides and high entropy MXenes are reviewed.
- High entropy materials as cathode, anode and electrolyte to improve batteries capacity, cycle life and cycle stability are introduced systematically.
- The latest progresses of employing machine learning in high entropy battery materials are highlighted and discussed in details.

## Graphical abstract

**Figure:**
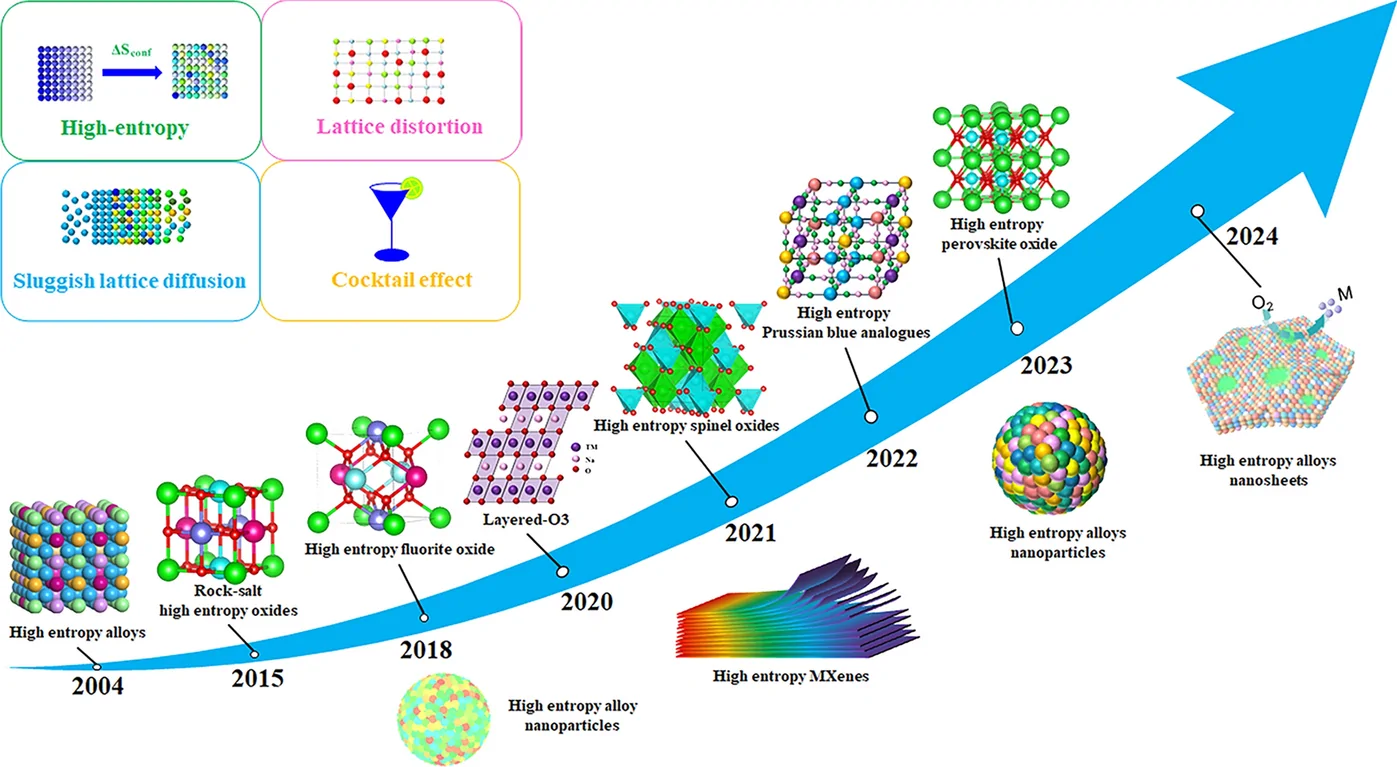

## Introduction

With the continuous growth of the global population and industrial development, energy demand is steadily increasing. However, fossil fuels such as coal and oil are finite resources [1]. As a result, renewable energy sources such as solar, wind, geothermal, and tidal energy have been extensively developed and deployed to address the limitations of finite fossil fuels. However, the intermittent and location-dependent nature of these energy sources necessitates the development of efficient and reliable energy storage systems. In this regard, batteries have emerged as indispensable components in modern energy infrastructures. Among them, lithium-ion batteries (LIBs), lithium-sulfur batteries (LSBs), sodium-ion batteries (SIBs), zinc-ion batteries (ZIBs), and potassium-ion batteries (PIBs) represent major research frontiers, each characterized by distinct electrode materials, electrolytes, and energy storage mechanisms. The electrochemical performance of these systems is closely linked to the properties and interactions of internal components. In particular, electrodes and electrolytes, as the core constituents of electrochemical energy storage systems, play a critical role in determining key performance metrics such as energy density, rate capability, and cycling stability. Consequently, substantial research efforts have been devoted to the development and optimization of diverse electrode materials and electrolyte systems [2–8]. Among the wide range of advanced electrode candidates, carbon-based materials have received considerable attention due to the structural tunability and favorable electrochemical characteristics. Among these, graphene, a two-dimensional (2D) crystalline material with a honeycomb-like bonding structure and a single atomic layer thickness, has attracted global attention due to its exceptional physical and chemical properties. The advantages of graphene-based materials lie in ability to enhance rate performance, increase capacity, and extend the lifespan of batteries. However, graphene composites are often hindered by poor initial Coulombic efficiency. In addition to carbon-based materials, transition metal oxides (TMOs) have also attracted significant interest due to high specific capacity. Nevertheless, TMOs often face challenges such as structural instability during cycling, which limits long-term performance in various battery systems. Beyond conventional batteries, air batteries have emerged as promising candidates for high-energy storage, relying heavily on efficient electrochemical reactions at the electrode interface. In this context, the development of catalytic electrode materials with high activity and stability is crucial to facilitate key reactions, including the oxygen reduction reaction (ORR), oxygen evolution reaction (OER), and hydrogen evolution reaction (HER), thereby improving the overall battery performance. Most catalytic materials currently employed in air batteries are precious metals, which, despite high catalytic activity, are costly and prone to generating undesirable intermediate species in alkaline environments. These drawbacks not only increase the overall cost but also adversely affect battery performance and durability. To address these issues, high-entropy materials (HEMs) have recently emerged as promising alternatives across diverse fields such as catalysis, materials science, and electromagnetics. By incorporating multiple principal elements, HEMs can significantly reduce the reliance on precious metals while maintaining or even enhancing catalytic activity. The broad elemental composition, coupled with the unique synergy between ordered crystal structures and compositional disorder, imparts exceptional multi-functional properties and improved catalytic performance. Therefore, HEMs are considered promising candidates for next-generation catalytic electrodes in advanced energy storage systems.

High-entropy alloys (HEAs, plural; HEA for singular) represent one of the earliest classes of HEMs, characterized by equimolar or near-equimolar incorporation of five or more principal elements. Compared with traditional alloys [9], HEAs exhibit superior mechanical properties, including high strength, hardness, wear resistance, and corrosion resistance, as well as excellent catalytic and thermoelectric properties. Building upon the success of HEA, other classed of HEMs, such as high-entropy oxides (HEOs, plural; HEO for singular) and high-entropy MXenes (HE MXenes; HE MXene for singular), have attracted growing interest, particularly in the fields of energy conversion and storage due to remarkable thermal stability, high ionic conductivity, and promising catalytic activity. Currently, research on HEMs encompasses material design, microstructure tuning, fabrication methods, microstructural characterization, mechanical properties, functional properties, and computational simulations [10–17]. As a result, HEMs have demonstrated substantial potential in a wide range of critical applications, including national defense, aviation, aerospace, and battery technologies [18–20].

In recent years, HEMs have garnered significant attention in the field of energy conversion and storage, particularly as emerging candidates for electrochemical energy storage systems. Owing to the unique structural characteristics and compositional complexity, HEMs have demonstrated the ability to enhance key battery performance metrics such as cycling stability and reaction kinetics. Moreover, effective solutions to the inherent limitations of conventional materials are offered, thereby accelerating advancements in battery technologies. As such, the exploration of HEMs carries substantial scientific importance and holds promising practical value. This review provides a comprehensive examination of recent developments concerning the application of HEMs in battery systems. It begins by outlining the historical trajectory of HEM research from 2004 to 2025, introducing foundational concepts and classifying major types of HEMs—namely, HEAs, HEOs, and HE MXenes. Subsequent sections detail the latest progress in employing HEMs as electrode and electrolyte materials in various battery systems, including LIBs, LSBs, zinc-air batteries (ZABs), ZIBs, SIBs, and PIBs. Compared with traditional electrode materials, the high-entropy-related effects significantly enhance the electrochemical performance of batteries, including capacity, cycling rate, and cycling stability. Benefiting from the inherent structural robustness and high ionic conductivity, HEMs also extend the electrochemical stability window, offering broader operational flexibility. Given the vast compositional diversity and tunability of high-entropy battery materials (HEBMs), there remains significant potential for performance optimization and functional innovation. To address the challenges of complex compositional design and property prediction, machine learning (ML) has recently emerged as an efficient strategy for accelerating the discovery and development of advanced HEMs. This review further highlights the integration of ML in the rational design of HEMs and discusses future research opportunities, with a particular focus on fabrication methodologies, structural and electrochemical characterization, and data-driven material optimization for next-generation energy storage systems.

### Historical Sketch

**Fig. 1:**
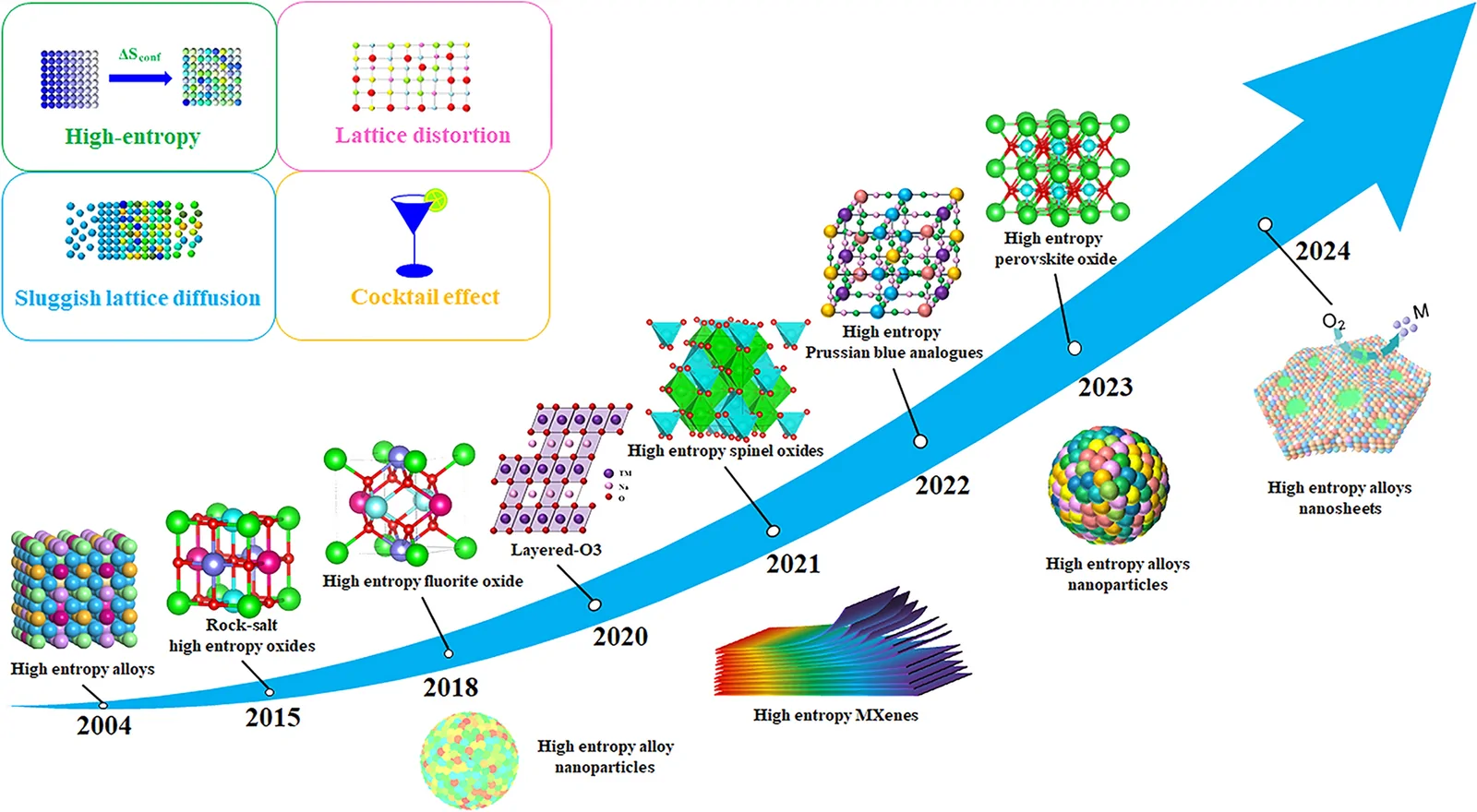
Four core effects and the historical overview of HEMs [24], Copyright 2024, Springer Nature

Since ancient times, humans have strived to develop new materials, discover new metals, and invent new alloys, such as iron and bronze. These alloys have traditionally been developed based on a “base element” model, typically starting with one and occasionally two principal elements. However, this paradigm began to shift with the emergence of multi-component alloy systems. The first detailed study of multi-component alloys, consisting of a large number of constituents in equal or near-equal proportions, was conducted in 1981 by Vincent [21]. This foundational work was later expanded by Cantor, who investigated novel multi-component amorphous alloys and explored the effects of high configurational entropy on alloy stability. Building on these insights, the first experimental results on crystalline multi-principal element alloys (MPEAs) were subsequently published, further establishing the theoretical and experimental basis for what would become known as HEAs [22]. In 2004, Yeh et al. [23] first formally proposed the concept of HEAs and defined them (Fig. 1). These unconventional structures offer opportunities to achieve unprecedented combinations of phase stability and mechanical performance, especially overcoming the strength–ductility trade-off. Following this conceptual breakthrough, research on HEAs expanded rapidly.

The hexagonal HEA was first reported in 2014 [25]. Until 2015, Rost et al. [26] applied the concept of high entropy to rock salt oxide (Mg_0.2_Co_0.2_Ni_0.2_Cu_0.2_Zn_0.2_O). Subsequently, Bérardan et al. [27] investigated the high Li^+^ ion mobility (> 10^–3^ S cm^−1^) and huge colossal dielectric constant in HEO, highlighting the potential for battery applications. In 2018, the HEA nanoparticles (HEA-NPs) were synthesized by the carbothermal-shock method, and the high-entropy fluorite oxides were prepared [28, 29]. In 2020, hollow HEO nanoparticles (HEO-NPs) were synthesized for the first time via the droplet-to-particle method [30]. Around the same time, high-entropy layered oxides began to emerge as promising cathode materials for SIBs [31], demonstrating the growing potential of HEMs in next-generation energy storage technologies. In 2021, Yao et al. [32] achieved a significant milestone by synthesizing HEA-NPs composed of up to 15 elements, setting a new benchmark for compositional complexity. In parallel, Anasori et al. [33] successfully synthesized high-entropy 2D MXenes for the first time by integrating the concepts of high-entropy compounds (HECs) and traditional MXenes architectures. In 2022, Ren et al. [34] employed laser powder bed fusion (L-PBF) to fabricate dual-phase nanolamellar HEAs (AlCoCrFeNi_2.1_), which exhibited an exceptional combination of high yield strength (~ 1.3 GPa) and large uniform elongation (~ 14%), outperforming conventional titanium alloys. These findings highlight the mechanical advantages of HEAs and inspire broader exploration of high-entropy strategies across functional materials. For example, the incorporation of high entropy into Prussian blue analogues (HE PBAs) as cathode materials for LSBs has received significant attention [35].

Previously, the synthesis of HEMs typically requires temperature exceeding 1,000 °C and complex processing techniques. However, recent breakthroughs have demonstrated the feasibility of more energy-efficient and scalable strategies. In 2023, Cao et al. [36] successfully synthesized HEA-NPs with multiple uniformly miscible elements under mild conditions via a liquid metal Ga-assisted route. This breakthrough demonstrates the feasibility of fabricating high-entropy structures without relying on extreme temperatures. Subsequently, Folgueras et al. [37] prepared high-entropy halide perovskite single crystals at room temperature using low-temperature solution processing. In the realm of electrocatalysis, the precise incorporation of single atoms into HEAs presents a promising approach to significantly enhance energy conversion efficiency. In 2024, He et al. [24] successfully synthesized single-atom Mo HEA nanosheets, exhibiting outstanding electrocatalytic performance for methanol oxidation reactions. Despite having a development history of just over two decades, HEMs have rapidly advanced and found broad applications, underscoring the increasing scientific and technological importance.

### Definitions and Concepts

HEMs are characterized by the absence of distinction between solvent and solute atoms. Yeh et al. [38] defined HEAs as those composed of five or more principal elements in equimolar ratios. To broaden the scope of alloy design, HEAs may contain principal elements with each element’s concentration ranging from 5 at% to 35 at% [23, 38]. Configurational entropy (*S*_*conf*_) is a physical quantity that quantifies the uncertainty or disorder in a mixed system. Additionally, HEAs can be defined using the *S*_*conf*_, as expressed in Eq. 1 [38]:

1 $$\it S_{{{\mathrm{conf}}}} = - R\mathop \sum \limits_{i = 1}^{N} x_{i} lnx_{i}$$

where *R* is the gas constant (*R* = 8.314 J K^−1^ mol^−1^), *N* is the number of elements, and *x*_*i*_ is the mole fraction of component *i*. Currently, it is generally accepted that MPEAs with a *S*_*conf*_ ≥ 1.5R are classified as HEAs. Materials with a *S*_*conf*_ between 1R and 1.5R are defined as medium entropy, while those with *S*_*conf*_ below 1R are considered low entropy. For HEOs, a system composed of equal molar ratios of components achieves the maximum mixed *S*_*conf*_ [39]. The *S*_*conf*_ of HEO systems, which contain both cations and anions, can be calculated using Eq. 2 [40]:

2 $$\it S_{{{\mathrm{conf}}}} = \left[ {\left( {\mathop \sum \limits_{i = 1}^{M} x_{i} lnx_{i} } \right)_{{\text{cation - site}}} + \left( {\mathop \sum \limits_{j = 1}^{N} x_{j} lnx_{j} } \right)_{{\text{anion - site}}} } \right].$$

where *M* and *N* present the number of cationic and anionic elements species, respectively. *x*_*i*_ and *x*_*j*_ are the mole fractions of ions at the cation and anion sites, respectively. It can be observed that *ΔS*_*conf*_ increases with the addition of more elements to a given system. There is evidence suggesting that as the number of elements increases, the *S*_*conf*_ also increases, leading to enhanced material stability [41]. However, for HEOs with multiple cation sites, such as high-entropy spinel oxides (HESOs, plural; HESO for singular, AB_2_O_4_) and high-entropy perovskite oxide (HEPOs, plural; HEPO for singular, ABO_3_), the calculation formula for *S*_conf_ is given by Eq. 3 [42]:

3 $$ \Delta S_{\mathrm{conf}} = \left[ {\left( {\mathop \sum \limits_{a = 1}^{M} x_{a} lnx_{a} } \right)_{\text{A cation - site}} + \left( {\mathop \sum \limits_{b = 1}^{O} x_{b} lnx_{b} } \right)_{\text{B cation - site}} + \left( {\mathop \sum \limits_{j = 1}^{N} x_{j} lnx_{j} } \right)_{\text{anion - site}}} \right]$$

Since accurately determining entropy values is challenging both experimentally and computationally, traditional approaches often approximate *S*_*conf*_ using mixing entropy (*S*_*mix*_). *S*_*mix*_ represents the entropy change associated with the formation of a mixture from different components. It is typically calculated using the ideal solution model or analogous models with similar assumptions [43]. Gibbs free energy universally quantifies the thermodynamic properties of all electrolyte systems, regardless of the diverse components. The formula for Gibbs–Helmholtz is given by Eq. 4 [44]:

4 $$\Delta G_{\mathrm{mix}} = \Delta H_{\mathrm{mix}} - T\Delta S_{\mathrm{mix}}$$

where the *ΔG*_*mix*_, *ΔH*_*mix*_, *ΔS*_*mix*_, and *T* represent Gibbs free energy, mixing enthalpy, mixing entropy, and reaction temperature, respectively. The *ΔS*_*mix*_ increases with the number of elements. By introducing multiple components to improve *ΔS*_*mix*_ or synthesizing at high temperature, phase stability can be achieved. At elevated temperatures, entropy becomes the dominant contributor to the total *ΔG*_*mix*_. Higher temperatures promote increased disorder among multi-component metal cations, facilitating the formation of new, simple metal oxide phases and resulting in more thermodynamically stable structures. It is important to note that *S*_*mix*_ is applicable only at high temperatures. An HEA remains stable only above a critical temperature; below this temperature—such as at room temperature—the solid solution may become unstable, leading to the formation of low-temperature multi-phase mixtures or intermetallic compounds. Cantor et al. [22] discovered through the study of Gibbs phase rule that FeCoMnNiCo, compared with other alloy compositions, can form a single-phase face-centered cubic (FCC) solid solution. Evans et al. [45] employed thermodynamic design approaches to visualize the transition of HEA solid solutions from high-temperature stability to metastable states, while identifying key thermodynamic characteristics associated with the persistence or decomposition of metastable HEAs.

In summary, reducing the *ΔG*_*mix*_ of the system and improving its stability, as described by the Gibbs–Helmholtz equation (Eq. 4), can be achieved by increasing the reaction temperature or the *S*_*mix*_. The *S*_*mix*_ can be enhanced by adding more elements. This strategy, which involves the incorporation of elements in equimolar or near-equimolar ratios, not only improves system stability but also leads to unexpected and unique effects. These effects are observed not only in HEAs but also in high-entropy ceramics, including HEOs, carbides, sulfides, and others [46–50]. As illustrated in the topleft side of Fig. 1, the underlying mechanisms can be attributed to four core effects of HEMs [39, 51–53]: Firstly, in thermodynamics, HEMs possess a high-entropy effect. At high temperatures, high *S*_*conf*_ can reduce the free energy of the solid solution phase, thereby promoting the formation of a stable phase. HEAs exhibit no distinction between solute and solvent atoms [53]. Secondly, due to the different radii of the constituent elements, severe lattice distortion effect can occur. However, lattice distortion can create more favorable reaction sites, thereby enhancing catalytic efficiency. Additionally, the high degree of disorder in HEMs provides a novel approach to controlling battery electrodes during the charging process. Thirdly, the sluggish lattice diffusion effect refers to the slower diffusion rate of individual elements in alloys with high *S*_*conf*_, compared to those with lower *S*_*conf*_. This phenomenon has the potential to provide significant thermal stability to several HEAs. Xiao et al. [54] demonstrated that Co can significantly influence the mutual diffusion coefficients of other major elements in HEAs, facilitating the preparation of HEAs with enhanced thermal stability. Lastly, the cocktail effect arises from the complex interactions of the individual units that compose the mixture, leading to unpredictable properties in the overall system. The strong synergistic effects between different elements, each with distinct physical properties, contribute to efficient energy conversion processes in HEMs. This is particularly evident in processes such as methanol oxidation, formic acid oxidation, OER, HER, and ORR [55–58].

### Thermodynamics Theory

The distinctive properties of HEMs stem from the multi-component composition. To maximize the solubility of components in single-phase mixtures, adherence to thermodynamic mixing rules is essential. In thermodynamics, the key factor enabling the formation of HEAs is the *S*_*conf*_ of the mixture. This principle facilitates the formation of high-entropy single-phase alloys. This effect is grounded in the second law of thermodynamics, which governs the direction of natural processes. According to this law, processes can either occur or not occur based on the spontaneity. Additionally, this principle is supported by the fundamental equations of thermodynamics, which predict that the equilibrium state corresponds to the lowest free energy state.

Many parameters are closely associated with the phase structure and element distribution of HEAs. In the crystal lattice of HEAs, significant differences in the size of component atoms can lead to severe lattice distortion. Therefore, studying the atomic size difference (*δ*) is crucial for selecting suitable alloy elements. The formula of *δ* can be expressed as follows [44]:

5 $$\delta =\sqrt{\sum_{i=1}^{n}{c}_{i}{\left(1-\frac{{r}_{i}}{{\overline{r} }_{i}}\right)}^{2}}$$

$${\overline{r} }_{i}=\sum_{i=1}^{n}{c}_{i}{r}_{i}$$

where *c*_*i*_ and *c*_*j*_ denote the atomic fraction and atomic radius of the *i*th element, respectively. *r*_*i*_ is the atomic radius. ${\overline{r} }_{i}$ is the average atomic radius of the elements participating in the alloy. *δ* is the important factor affecting the lattice distortion of HEAs. The mixing enthalpy can be calculated using Eq. 6 [44]:

6 $${H}_{mix}=\sum_{i-1, i\ne j}^{n}4\Delta {H}_{ij}^{mix}{c}_{i}{c}_{j}$$

where *c*_*i*_ and *c*_*j*_ are the atomic percentages of elements *i* and *j*, respectively. And $\Delta {H}_{ij}^{mix}$ is the enthalpy of the binary liquid state of elements *i* and* j* in an equiatomic composition.

In the study of whether a mixture can form a solid solution, the parameter *Ω* is introduced. If *Ω* equals 1, the entropy contribution to the mixture’s temperature will exceed the enthalpy contribution, favoring the formation of solid solutions in HEAs. Conversely, when *Ω* is less than1, the enthalpy of the mixture becomes the dominant factor. The formula for *Ω* can be expressed as follows in Eq. 7 [59]:

7 $$\Omega =\frac{{T}_{\mathrm{m}}{\Delta S}_{\mathrm{mix}}}{|{\Delta H}_{\mathrm{mix}}|}$$

$${T}_{\mathrm{m}}={\sum }_{i=1}^{n}{c}_{i}{T}_{{\mathrm{m}}_{i}}$$

where *T*_*m*_ is the melting temperature calculated by the mixing rule, ${T}_{{\mathrm{m}}_{i}}$ is the temperature of the melting point of the element, and *c*_*i*_ represents the atomic fraction of the element each element.

The valence electron refers to the electrons that are involved in interactions with other atoms to form chemical bonds. The valence electron concentration (*VEC*) of HEAs is closely related to the structural stability, as it influences the characteristics of bonding and atomic stacking. The *VEC* can be calculated using the following equation [60]:

8 $$VEC=\sum_{i=1}^{n}{c}_{i}{(VEC)}_{i}$$

where *(VEC)*_*i*_ and *c*_*i*_ represent the *VEC* and the percentage of atoms of the *i*th component, respectively.

Electronegativity represents the preference of atoms for electrons. The difference in the Pauling electronegativity (*Δχ*_*Pauling*_) of HEAs is defined as follows [60]:

9 $$\Delta {\chi }_{\mathrm{Pauling}}=\sqrt{\sum_{i=1}^{n}{c}_{i}{({\chi }_{i}-{\chi }_{avg})}^{2}}$$

$${\chi }_{avg}=\sum_{i=1}^{n}{c}_{i}{\chi }_{i}$$

where *χ*_*i*_ and *c*_*i*_ represent the Pauling electronegative and the atomic percentage of the *i*th component, respectively. Understanding the significance of each parameter is crucial for the effective design of HEAs that meet specific performance expectation.

## Classification and Characteristics of HEMs

HEMs encompass HEAs, HEOs, HE MXenes, and other compounds. Since the first report on HEAs in 2004 [61], this field has experienced rapid advancement. The high-entropy effect has enabled HEAs to overcome the conventional trade-off between strength and toughness, surpassing traditional alloys in both aspects. Beyond basic metals, HEAs have involved to include refractory high-entropy alloys (RHEAs), further broadening the HEA system [62–64]. Meanwhile, the advent of HEOs has opened new avenues for the application of HEMs, leading to the realization of additional HECs, such as high-entropy sulfides [65], carbides [66, 67], and MXenes [33, 68, 69]. In addition, various structures of HEOs, including rock salt type [70], spinel type [18, 71, 72], perovskite-type [37, 73–75], and O3/P3/P2 layered HEOs [76–80], have also been investigated. These diverse structures are closely related to the intrinsic characteristics of HEMs. The key effects—high entropy, slow diffusion, and severe lattice mismatch—have drawn significant attention in the field of batteries, particularly for HEOs. Additionally, the use of high-entropy nanoparticles has been extensively explored in electrocatalysis. To date, HEMs have been successfully employed as cathode, anode, and electrolytes in batteries, demonstrating remarkable cycling performance and high specific capacity [81–92].

### HEAs

**Fig. 2:**
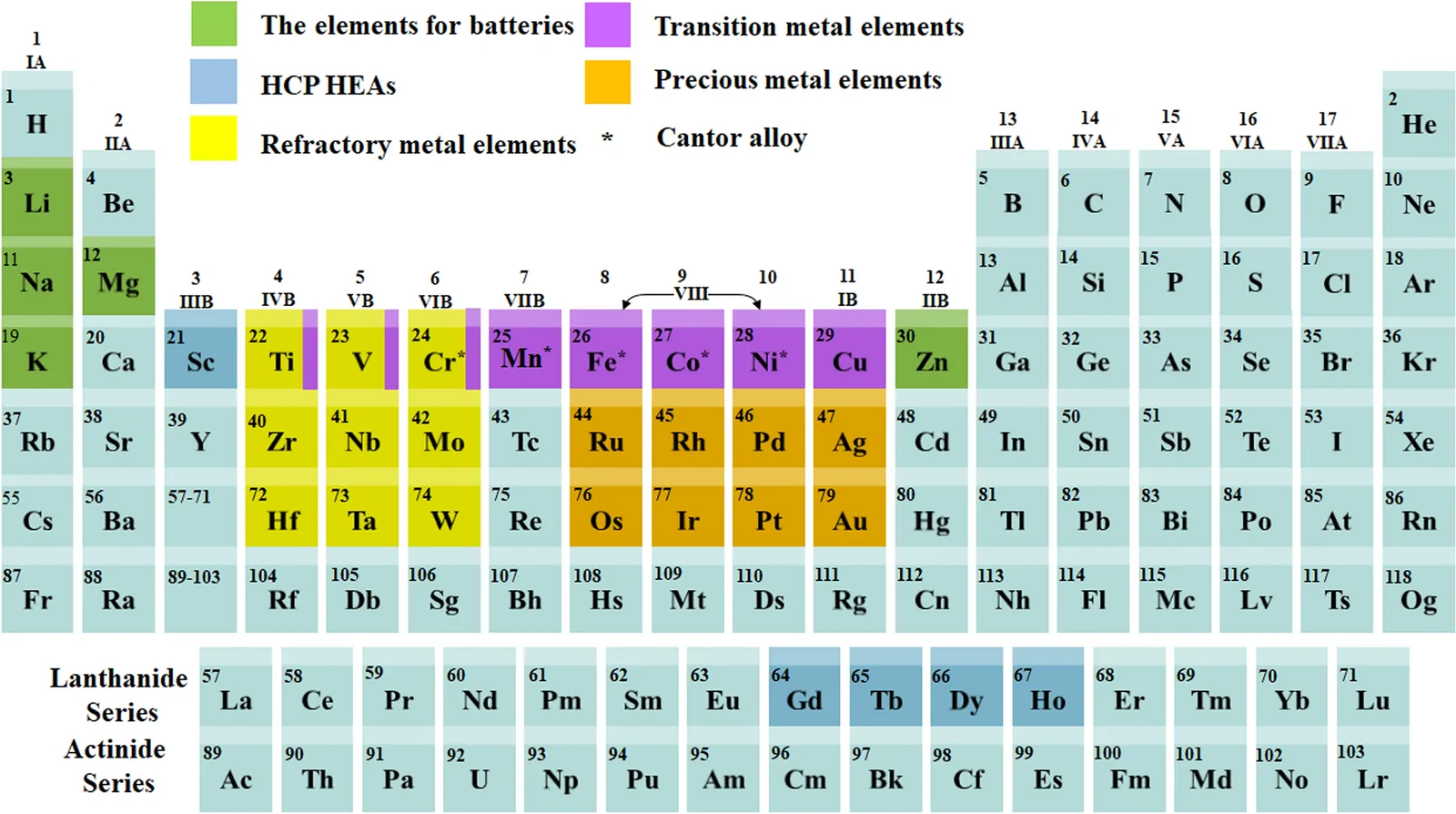
Possible crystal structures of different elements in HEMs

Since the concept of HEAs was first proposed in 2004, the field has experienced steady development in its early years, with foundational studies laying the theoretical and experimental groundwork. Around 2013, the number of related journal publications began to rise sharply, marking a pivotal turning point that signaled widespread recognition and growing research interest. Over the past two decades, more than 10,000 articles on HEAs have been published, accumulating over a million citations, which highlights the increasing importance of HEAs as a vibrant and influential research topic in materials science [61]. HEAs are situated near the center of the multi-component phase diagram, whereas traditional alloys typically focus on the corners or edges of the system [21]. HEAs are composed of five or more metal elements selected from the periodic table, as illustrated in Fig. 2. Initially HEAs consisted of five equimolar elements but have gradually evolved to include alloys containing 15 or more elements [32]. HEAs with a single-phase crystal structure have been synthesized using various physical and chemical methods, including arc melting, ball milling, additive manufacturing (AM), solvothermal synthesis, and ultra-assisted wet chemistry. Additionally, techniques such as carbothermal shock [28], liquid-phase reduction [36, 93], and fast-moving bed pyrolysis [94, 95] have been employed to prepare HEA-NPs. To date, HEA-NPs have been synthesized under low and mild temperature conditions [36].

Beyond the versatile synthesis methods, HEAs also exhibit remarkable mechanical properties. For traditional alloys, there is often a trade-off between strength and ductility. However, HEAs can simultaneously enhance both strength and ductility, and prolong strain hardening, by controlling deformation mechanisms such as edge dislocation motion, stacking fault formation, nano-twinning, and phase transformation. For instance, Lei et al. [96] employed ordered oxygen complexes in HEAs to achieve significant improvements in tensile strength and ductility. Pan et al. [97] introduced a gradient cell structured material into HEA, which substantially increased the yield strength while maintaining good plasticity. Ding et al. [98] innovatively replaced Ni with Pd in Cantor alloy (FeCrMnNiCo), observing a cross-slip phenomenon at room temperature, which resulted in enhanced yield strength of the alloy. Furthermore, the excellent strength of body-centered-cubic (BCC) HEAs (MoNbTaVW) at temperatures up to 1900 K is primarily attributed to the movement of edge dislocations in random alloys. RHEAs, a new class of HEAs composed of high-melting-point metals, have garnered significant attention in recent years due to remarkable potential as high-temperature structural materials [99]. Wang et al. [100] modified the BCC structure of the TiZrHfNb prototype HEA by reducing Nb content and lowering the deformation temperature, which activated mechanical twinning and substantially improved strain hardening behavior and the combination of strength and plasticity. The investigation of twinning-induced plasticity in RHEAs provides valuable insights into the deformation mechanisms of multi-component refractory systems, aiding the design of high-performance RHEAs. Cui et al. [101] achieved phase stability, high tensile yield strength, and excellent elongation through the controlled doping of oxygen in RHEA (Ti_41_V_27_Hf_15_Nb_15_O_2_).

Despite the remarkable strength and thermal stability of RHEAs at elevated temperatures, a critical challenge persists regarding room-temperature brittleness, which limits competitiveness with traditional high-temperature alloys. The in-situ formation of a heterogeneous dual-phase structure has been proposed as a means to mitigate this brittleness. Compared to traditional processing methods, AM offers the capability to directly fabricate complex parts. AM enables new opportunities for producing geometrically intricate HEAs, allowing in situ tailoring of microstructural features. Examples include HEAs with dual-phase nanolayers, RHEAs exhibiting exceptional strength and plasticity, and HEAs with featuring nano-bridged honeycomb microstructures [34, 102, 103]. Annealing, growth, and deformation twinning are critical processes that significantly influence the mechanical properties of materials during the preparation of HEA. From an engineering perspective, a thorough understanding of the key parameters governing the microstructure of twinned materials is essential [104].

#### Fundamental Properties of HEAs

HEAs, characterized by the well-established four core effects, not only exhibit an exceptional balance between strength and ductility at room temperature but also deliver excellent performance in extreme environments, ranging from cryogenic to ultra-high temperatures. At low temperatures, HEAs demonstrate remarkable fracture resistance, which is primarily attributed to the activation of planar slip dislocations [105]. Moreover, HEAs offer significant advantages in several other properties, including elasticity [106], high-temperature damping performance [107, 108], soft magnetism [109, 110], radiation resistance [111], corrosion-resistance [112], and wear resistance [113, 114]. Currently, HEAs are classified using two primary approaches: (1) By elemental composition, HEAs can be divided into 3d transition metal (TM) HEAs, RHEAs, rare earth HEAs, precious metal HEAs, and non-metallic element-doped HEAs; (2) By functional characteristics and application areas, HEAs are categorized lightweight HEAs, high-temperature HEAs, RHEAs, corrosion-resistant HEAs, radiation-resistant HEAs, biomedical HEAs, eutectic HEAs, wear-resistant HEAs, hydrogen storage HEAs, catalytic HEAs, and soft magnetic HEAs. In addition to compositional and functional diversity, HEAs also exhibit a wide range of structural forms, including, including BCC, FCC, and hexagonal close-packed (HCP) [23, 113, 115]. Among these, the FCC structure tends to be thermodynamically stable at high temperatures, whereas the HCP structure becomes more favorable under low-temperature conditions [116]. Building upon the diversity of crystal structures in HEAs, these alloys can also be classified according to the phase types into four categories: single-phase, dual-phase, common-phase, and multi-phase systems.

**Fig. 3:**
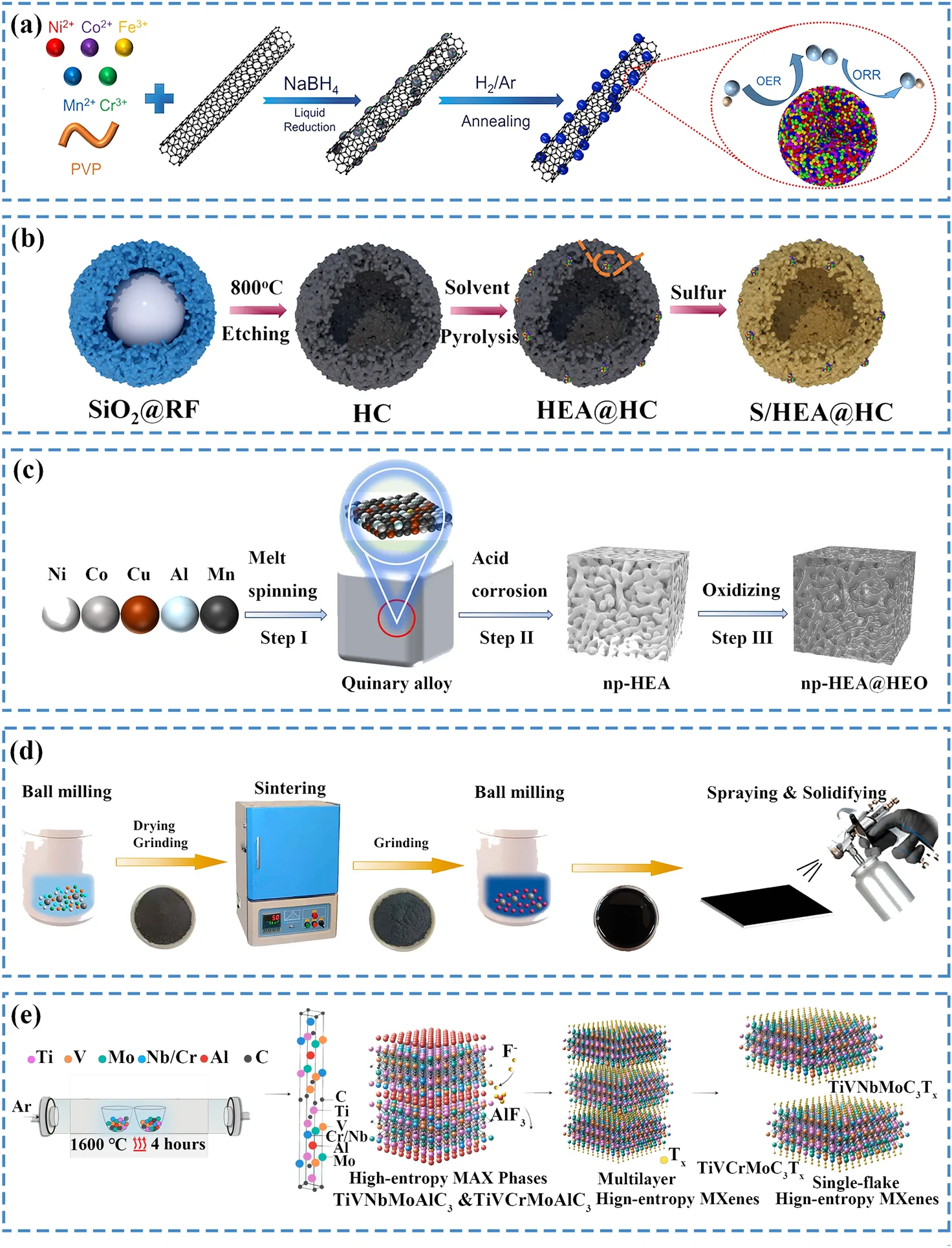
Schematic diagram of preparation process of **a** Fe_12_Ni_23_Cr_10_Co_55-*x*_Mn_*x*_/CNT and principle of bifunctional ORR/OER [93], Copyright 2023, American Chemical Society. **b** S/HEA@HC [117], Copyright 2024, American Chemical Society.** c** np-HEA@HEO [119], Copyright 2023, Elsevier. **d** Ball milling process of (Cu, Mn, Fe, Cr)_3_O_4_ [121], Copyright 2022, American Chemical Society.** e** HE MAX and MXenes [33], Copyright 2021, American Chemical Society

There are various methods for preparing HEAs, such as HEA-NPs, hollow HEA-NPs, dual-phase nanolamellar HEAs, HEA ultra-thin nanosheets, and RHEAs. These include techniques such as the carbothermal shock [28], melt spinning [22], electromagnetic levitation melting [25], droplet-to-particle [30], L-PBF [34], liquid-phase reduction [93], bed pyrolysis strategy [95], arc-melting [98], AM [108], magnetron sputtering [111], impregnation method [117], vacuum induction melting [118], as shown in Table 1. Different synthesis methods produce HEAs in various forms where arc melting, L-PBF and AM are used for bulk HEAs while carbothermal shock, fast-moving bed pyrolysis, melt spinning, droplet-to-particle conversion and electromagnetic levitation melting prepare HEA powders with AM and magnetron sputtering being suitable for HEA films and coatings. For example, Cao et al. [93] obtained carbon nanotube (CNT) HEA through liquid-phase reduction, as shown in Fig. 3a. Separately, Han et al. [117] synthesized Pt_0.25_Cu_0.25_Fe_0.15_Co_0.15_Ni_0.2_ HEAs on hollow carbons supports using an impregnation method (Fig. 3b). Meanwhile, Wei et al. [119] prepared HEA containing Ni, Co, Cu, Al, and Mn via electromagnetic levitation melting (Fig. 3c).

**Table 1:** The structure and preparation method of HEAs

Composition	Structure	Method	Refs
PtPdRhRuCe	Nanoparticle	Carbothermal shock	[28]
Fe_20_Cr_20_Mn_20_Ni_20_Co_20_	FCC	Melt spinning	[22]
Ho-Dy-Y-Gd-Tb	Hexagonal	Electromagnetic levitation melting	[25]
RuIrFeCoNi	Hollow nanoparticles	Droplet-to-particle	[30]
AlCoCrFeNi_2.1_	FCC + BCC	L-PBF	[34]
Fe_12_Ni_23_Cr_10_Co_55-*x*_Mn_*x*_	FCC	Liquid-phase reduction	[93]
MnCoNiCuRhPdSnIrPtAu	Nanoparticles	Fast-moving bed pyrolysis	[95]
CrFeCoNiPd	FCC	Arc-melting	[98]
CrMnFeCoNi	FCC	Arc-melting	[105]
Fe_48_ Co_10_ Cr_10_ Mn_32_	FCC + HCP	Arc-melting	[107]
Cr_20_Mn_6_Fe_34_Co_34_Ni_6_	FCC + HCP	AM	[108]
W-Ta-Cr-V-Hf	BCC	Magnetron sputtering	[111]
Pt_0.25_Cu_0.25_Fe_0.15_Co_0.15_Ni_0.2_	FCC	Impregnation method	[117]
Fe_50_Mn_30_Co_10_Cr_10_	FCC + HCP	Vacuum induction furnace	[118]
Al_0.1_CoCrFeNi	FCC	Arc melting	[120]

#### Applications of HEAs

Over the past two decades, HEAs have been demonstrated a wide array of outstanding properties, including exceptional specific strength [63, 97, 101, 118], high wear resistance [113, 114], remarkable tensile ductility [120], superior mechanical properties at elevated temperatures [122], and excellent fracture toughness at cryogenic temperatures [123]. In addition to mechanical advantages, HEAs also exhibit functional properties such as remarkable superparamagnetism [124], superconductivity [125, 126], and high electrocatalytic activity [30, 127, 128]. Owing to these unique characteristics, HEAs have attracted increasing interest across a wide range of fields, including aerospace, navigation, energy, chemical engineering, national defense, electronics, nuclear energy, surface engineering, batteries, and electrocatalysis. In particular, the energy sector has seen growing attention toward the functional potential of HEAs in clean and sustainable technologies. For instance, due to the severe lattice distortion and the presence of abundant favorable reaction sites, HEAs have emerged as promising candidates for hydrogen storage. In addition, RHEAs, a class of HEAs composed of high-melting-point elements, not only demonstrate excellent high-temperature performance but also exhibit ultra-high tensile yield strength and good ductility even at room temperature—highlighting a remarkable synergy between strength and plasticity [63, 64, 100]. Building on these strengths, HEAs with high thermal stability are well-suited for demanding applications in aerospace, automotive, and nuclear engineering. Furthermore, high-strength, high-toughness dual nanostructured HEAs fabricated through additive manufacturing techniques such as 3D printing show great promise for deployment in energy, biomedical, aerospace, and transportation industries [129].

On the one hand, HEAs have demonstrated great potential as catalysts for energy conversion [130]. For example, an (IrRuNiMoCo) HEA with an Ir-rich medium-entropy oxide (Ir-MEO) shell (HEA@Ir-MEO) has been shown to enhance acidic OER [131]. HEAs with different structures are employed as anode or cathode materials in batteries to improve cycling stability and specific capacity. Moreover, the high-entropy intermetallic (HEI) i-(PtPdIrRu)_2_FeCu effectively inhibits strongly absorbed carbon monoxide intermediates during formic acid oxidation reactions [132]. HEA-NPs have also been identified as efficient catalysts for alkaline overall seawater splitting [133]. HEA nanosheets and single-atom Mo-tailored HEA ultra-thin nanosheets have been used to fine-tune the hydrogen evolution and methanol oxidation reactions, respectively [24]. CNTs possess long-range ordered porous properties and high conductivity, facilitating the accommodation of more electrochemical materials [134, 135]. When HEAs are loaded onto CNTs, excellent HER and OER performance in alkaline seawater is demonstrated [133].

On the other hand, HEAs have broad applications in energy storage such as LIBs [136], LSBs [137], H_2_ batteries [58], ZABs [93, 133, 138], and Li-O_2_ batteries [139, 140]. For example, the integration of HEAs with Si effectively suppresses phase separation in Si, Al, Mg, and Ge systems through entropy stabilization mechanisms, thereby mitigating internal stress and maintaining structural integrity. This composite system demonstrates exceptional electrochemical performance in LIBs, delivering a high specific capacity of 2200 mAh g^−1^ while maintaining 94.6% capacity retention after 50 cycles [136]. Moreover, HEAs exhibit exceptionally sluggish diffusion behavior and low *ΔG*_*mix*_, which endow them with superior oxidation resistance and chemical stability. As a result, when nano-HEA was employed to modify separator for LSBs, an initial discharge capacity of 816 mAh g^−1^ was achieved, with a retained capacity of 680 mAh g^−1^ after 500 cycles, demonstrating an impressive capacity retention rate of ~ 83.3% and an average Coulombic efficiency exceeding 99% [137]. Furthermore, HEAs have emerged as highly efficient bifunctional ORR/OER catalysts for ZABs, outperforming conventional RuO_2_ and Pt/C catalysts. The exceptional catalytic performance stems from multiple synergistic effects including twin defect formation, substantial lattice distortion, and strong electronic interactions between constituent elements. When configured as air cathode for ZABs, these HEAs demonstrate outstanding electrochemical characteristics: an open-circuit voltage reaching 1.489 V, peak power density of 116.5 mW cm^−2^, specific capacity as high as 836 mAh g^−1^, and exceptional operational stability maintaining performance for over 10 days of continuous cycling [141]. In addition, the MnCoNiCuZn-based nanoparticles encapsulated in nitrogen-doped carbon matrices demonstrate exceptional performance as anodes for PIBs, achieving remarkable cycling stability exceeding 3,000 cycles while delivering impressive specific capacity (513 mAh g^−1^) and outstanding rate capability (202 mAh g^−1^ at 5 A g^−1^). Through combined ex-situ characterization and density functional theory (DFT) calculations, these HEA-NPs are revealed to function as atomic composites, forming interstitial metallic solid solutions with potassium through synergistic interactions between the constituent elements [142].

### HEOs

Inspired by advances in metal alloys research and ground in fundamental principles of thermodynamics, Rost et al. [26] extended the concept of entropy to five-component oxides, leading to the discovery of the first entropy-stabilized material with rock-salt structure. Subsequently, Bérardan et al. [27] demonstrated that Li and Na-substituted HEOs exhibit remarkably high ionic conductivity, highlighting potential for Li or Na batteries. Sarkar et al. [143] revealed that Co^2+^ and Cu^2+^ ions actively participate in the transformation reactions within the rock-salt structure, whereas other cations primarily serve to stabilize the lattice, acting as a structural matrix. Meanwhile, Mg^2+^ and Ni^2+^ ions help preserve structural integrity during oxidation, contributing to the overall stability of the material.

Today, HEOs are extensively employed as anodes, cathodes, and electrolytes in Li, Na, Zn, and K batteries, as well as in fuel cells. High-entropy metal oxides (HEMOs), in particular, serve as effective chemical anchors for polysulfides in LSBs [144]. Owing to the synergistic effects of Li–O and Ni–S bonds, HEOs can promote the ORR of lithium polysulfides (LiPSs) and mitigate the shuttle effect between the cathode and anode [12]. Furthermore, HEO-NPs have also attracted significant attention as catalysts and supercapacitor materials due to the multi-element composition and unique high-entropy mixing state, which together enhance both catalytic activity and structural stability [145]. Beyond rock-salt and spinel-type high-entropy oxides (HESOs), various other HEO systems have emerged, including TMOs [146], sodium superionic conductors (NASICON)/ lithium superionic conductors (LISICON) [147–150], HEPOs [11, 73, 151], PBAs [35], and garnet structure compounds [152], all of which exhibit remarkable enhancements in both mechanical robustness and electrochemical performance.

#### Fundamental Properties of HEOs

HEOs, also known as entropy-stabilized oxides, have *S*_*conf*_ of HEOs calculated using Eqs. 2 and 3. The unique multi-anion oxide structure combined with high-entropy characteristics gives rise to four core effects in HEOs. Consequently, compared to traditional oxide materials [153, 154], HEOs demonstrate superior properties, including excellent mechanical strength, enhanced structural and high-temperature stability, as well as a notable magnetocaloric effect. Various structures of HEOs have been synthesized, including rock-salt [70], spinel [18, 50, 71, 72, 121, 145, 155–166], perovskite [11, 37, 55, 56, 73–75, 84, 151, 167–173], fluorite [29, 174, 175], O3/P2/P3 layered oxide [76–80, 176], and PBAs [35, 177, 178]. Relaxor ferroelectrics are crucial in technological applications due to the strong electromechanical response, energy density, electrocaloric effect, and pyroelectric energy conversion properties [179, 180].

From the perspective of preparation methods, various HEOs with different structures have been successfully fabricated through diverse approaches, as summarized in Table 2. In general, these methods exhibit significant differences in regulating key material performance indicators, such as grain boundary distribution and oxygen vacancy concentration. Therefore, Table 3 systematically compares mainstream methods based on four critical dimensions: (1) material growth uniformity, (2) grain size control, (3) structural compatibility, and (4) process energy consumption, along with associated challenges. Specifically, HEOs with rock-salt, spinel, and layered structures are predominantly synthesized via solid-state reaction due to its process simplicity. However, this method often faces challenges such as phase separation and high energy consumption. For nanoparticle or thin film fabrication, the sol–gel method is preferred, despite its reliance on expensive metal–organic precursors. Meanwhile, co-precipitation offers an energy-efficient alternative for producing rock-salt and spinel HEO-NPs, while hydrothermal synthesis enables low-temperature formation of layered HEOs with controlled crystallinity. Ball milling has emerged as the primary technique for fluorite-structured HEOs through mechanical alloying. Figure 3d illustrates the fabrication process of HEOs via solid-state reaction combined with ball milling method [121]. Additionally, when 1D nanostructures are required for flexible electrodes or catalyst supports, electrospinning stands out as the optimal choice due to its ability to precisely tune fiber morphology and porosity. In summary, each synthesis method presents distinct advantages and limitations that must be carefully considered based on the target HEOs structure and intended application.

**Table 2:** The structure and preparation method of HEOs

Composition	Structure	Method	Refs
(Cr_0.2_Mn_0.2_Fe_0.2_Co_0.2_Ni_0.2_)_3_O_4_	Spinel	Solid-state reaction	[18]
Mg_0.2_Co_0.2_Ni_0.2_Cu_0.2_Zn_0.2_O	Rock-salt	Solid-state reaction	[26]
(Hf_0.2_Zr_0.2_Ce_0.2_)(Yb_0.2_Gd_0.2_)O_2-*δ*_	Fluorite	Ball milling	[29]
CoNiCuMnZnFe-PBA	PBA	Co-precipitation	[35]
(MnFeCoNiCr)_3_O_4_	Spinel	Hydrothermal	[72]
(La_*x*_K_0.4−*x*_Ca_0.2_Sr_0.2_Ba_0.2_)- TiO_3+*δ*_	Perovskite	Solid-phase reaction	[73]
Na(Fe_0.2_Co_0.2_Ni_0.2_Ti_0.2_Sn_0.1_Li_0.1_)O_2_	Layered	Solid-state reaction	[76]
NaNi_0.12_Cu_0.12_Mg_0.12_Fe_0.15_Co_0.15_Mn_0.1_Ti_0.1_Sn_0.1_Sb_0.04_O_2_	Layered	Solid-state reaction	[82]
Mg_0.2_Co_0.2_Ni_0.2_Cu_0.2_Zn_0.2_O	Rock-salt	Nebulized spray pyrolysis	[88]
Pd_1_-(CeZrHfTiLa)O_*x*_	Fluorite	Mechanical milling	[175]
(CrNiMnFeCu)_3_O_4_	Spinel	Facile hydrothermal	[181]
Na_3_V_1.9_(CaMgAlCrMn)_0.1_(PO_4_)_2_F_3_	Fluorophosphate	Solid-phase reaction	[182]
Li_0.20_Mg_0.16_Co_0.16_Ni_0.16_Cu_0.16_Zn_0.16_O	rock-salt	Sol–gel	[183]
Mg_0.2_Co_0.2_Ni_0.2_Cu_0.2_Zn_0.2_O	Rock-salt	Solid-state reaction	[184]
La-(Mn_0.2_Cu_0.2_Co_0.2_Ni_0.2_Fe_0.2_)O_3_	Perovskite	Electrospinning	[185]

**Table 3:** A systematic comparison of the key characteristics of mainstream HEOs synthesis methods, with particular emphasis on distinct features and primary challenges

Methods	Characteristics of the Method	Main challenges
Solid-state reaction	Uniformity: Moderate Particle Size: Micron-scale Applicable Systems: Rock-salt, Layered or spinel Energy Consumption: High	Challenges: Phase separation and high energy consumption
Sol–gel	Uniformity: High Particle Size: Nanoscale Applicable System: Rock-salt Energy Consumption: Medium	Challenges: High precursor cost and gel shrinkage
Co-precipitation	Uniformity: Medium–high Particle Size: Nanoscale Applicable Systems: Rock salt or Spinel Energy Consumption: Low	Challenges: Susceptibility to agglomeration
Hydrothermal	Uniformity: High Particle Size: Nano- to submicron-scale Applicable Systems: Spinel, Layered or Core–shell Energy Consumption: Low	Challenges: Extended reaction times and high equipment cost
Ball milling	Uniformity: Moderate Particle Size: Nanoscale Applicable System: Fluorite Energy Consumption: Moderate	Challenges: Potential contamination hazards
Electrospinning	Uniformity: High Particle Size: Nanofiber Applicable System: 1D structure Energy Consumption: Medium	Challenges: Low fiber strength and susceptibility to breakage

#### Applications of HEOs

HEOs have found widespread applications in various aspects of batteries, including cathodes, anodes, and electrolytes. Notable applications of HEOs include LIBs [162, 181, 186–189], LSBs [35, 75, 144, 190–192], Li-metal batteries [193], aqueous zinc-ion batteries (AZIBs) [194], ZABs [195], SIBs [76–79, 82, 148, 177, 182, 196–203], and PIBs [86, 202]. In LIBs, the HESOs have demonstrated excellent anode performance, delivering 865 mAh g^−1^ with 90% capacity retention over 200 cycles [164]. Moreover, the interfacial engineering of surface-chemically modified HEO@polyaniline heterojunctions anode exhibits remarkable rate capability (325 mAh g^−1^ at 10 A g^−1^) while maintaining 822.7 mAh g^−1^ reversible capacity [204]. Owing to the synergistic effect of lattice distortion and oxygen vacancies, the perovskite-structured Gd(Co_0.2_Cr_0.2_Fe_0.2_Mn_0.2_Ni_0.2_)O_3_ serves as an effective anode material, exhibits superior high-rate lithium-ion storage performance (300 mAh g^−1^) and excellent cycling stability (394 mAh g^−1^ after 500 cycles) [11]. Transitioning to SIBs, O3-type cathodes provide 111 mAh g^−1^ with 81.4% retention after 100 cycles [76], while layered NaNi_0.2_Fe_0.2_Mn_0.35_Cu_0.05_Zn_0.1_Sn_0.1_O_2_ exhibits capacity of 128 mAh g^−1^ with 87% retention after 500 cycles [198]. The P2/O3 biphasic Na_0.75_Cu_0.1_Fe_0.2_Mg_0.2_Mn_0.4_Ti_0.1_O_2_ cathode combines high capacity (167.8 mAh g^−1^) with stable cycling (86.7% retention after 100 cycles) [205]. The electrochemical behavior of HEOs exhibits significant variations between LIBs and SIBs, primarily due to differences in ion transport kinetics and interfacial stability. Compared to SIBs, HEOs exhibit superior capacity, rate capability, and cycling performance in LIBs. The enhanced performance of HEOs in LIBs stems from fundamental advantages: the small size and weak polarizability of Li^+^ ions are better accommodated within the complex structure of HEOs, enabling faster diffusion kinetics, more stable lattice frameworks, and more efficient interfacial reactions. In contrast, the larger ionic radius and thermodynamic limitations of Na^+^ lead to significantly degraded performance in the same materials. To address these challenges, future improvements for SIB applications may focus on targeted design strategies, such as expanding interlayer spacing or introducing sodium-active sites to enhance ion transport and stability.

In HEOs, elements such as Cr, Mn, Fe, Co, and Ni exhibit promising redox behavior and favorable charge storage properties. The heteronuclear coordination between single-atom Ru and HEPO facilitates rapid electron transfer from Ru to HEPO by forming Ru–O–M (where M represents Mn, Co, Fe, or Ni) bridges. This process effectively redistributes electrons within the Ru@HEPO complex, significantly enhancing interfacial charge transfer kinetics and improved electrocatalytic activity [55]. Moreover, HEO (Mg_0.2_Co_0.2_Ni_0.2_Cu_0.2_Zn_0.2_O), where the Cu–Co pair exhibits a high-spin Co configuration, is also utilized for the electrochemical reduction of ammonia with nitric acid, demonstrating state-of-the-art catalytic performance [183].

### HE MXenes

The MAX phase was first defined by Barsoum in 2001 [206]. The chemical formula of the MAX phase is M_*n*+1_AX_*n*_ (*n* = 1,2,3), where M represent an early transition-metal element, A is an element predominantly from groups 13–16 (Fig. 2), and X is C and/ or N. MXenes (MXene for singular) are derived by selectively etching the A-layer, which contains relatively weak bonds in the 3D MAX phase. Consequently, the chemical formula of MXenes changes to M_*n*+1_X_*n*_T_*x*_ (T_*x*_ = -F, -O, -OH, -Cl, and *n* = 1–4). Depending on the value of n, the metal atoms exhibit different ordering. For *n* = 1, an ABABAB stacking sequence is observed, while MXenes with *n* ≥ 2 exhibits ABCABC ordering [207].

MXenes, a large family of 2D early TM carbides and carbonitrides, were first discovered in 2011 [208]. Known for the unique hydrophilicity, conductivity, and redox pseudocapacitance, MXenes have garnered significant attention in the field of energy catalysis. However, the performance of traditional 2D MXenes is limited due to constrained interlayer spacing and structural instability. In 2021, Anasori et al. [33] synthesized high-entropy 2D MXenes for the first time, inspired by HECs and 2D MXenes. Subsequently, the chemical order and disorder of TiVNbMoC_3_ and TiVCrMoC_3_ were predicted using high-throughput first-principles calculations combined with DFT, cluster expansion, and Monte Carlo simulations. It was found that Cr preferentially occupies the outer layer, followed by Mo, V, Nb, and Ti. However, at lower temperatures, Nb and V in TiVNbMoC_3_ tend to segregate [10]. To date, six compositions of HE MXenes have been reported: M_*n*_C [67], M_*n*_C_2_ [209, 210], M_*n*_C_3_ [33], M_*n*_CT_*x*_ [211], M_*n*_C_2_T_*x*_ [69, 212], and MnC_3_T_*z*_ [213], where n represents the number of early TMs (*n* = 4, 5). The concept of HE MXenes has opened up broad prospects for applications in catalysis, energy storage, electromagnetic shielding, biomedical fields, and superconductivity. The synthesis of high-entropy MAX (HE MAX) phases and MXenes involves three critical steps: (1) stoichiometric mixing and sintering of TM powders (Ti, V, Nb, Cr, Mo), Al, and C to form layered M_4_AlC_3_ MAX phases; (2) selective etching of Al layers using hydrofluoric acid to produce multi-layer MXene M_4_C_3_T_*x*_; and (3) organic intercalation-assisted delamination to obtain single-layer MXenes (e.g., TiVNbMoC_3_T_*x*_), as shown in Fig. 3e [10]. Currently, the most common method for synthesizing HE MXenes is selective etching of HE MAX phases. This approach offers moderate uniformity in product morphology while maintaining the characteristic layered particle structure. The technique is specifically applicable to MAX phase precursor systems, where the A-layer elements are selectively removed through chemical etching. However, this method presents significant challenges, including high energy consumption due to the elevated temperatures required for effective etching, and the inevitable introduction of structural defects during the etching process. These defects may include vacancy formation, surface functional group variations, and structural distortions in the resulting MXene layers, which can impact the material’s electronic properties and mechanical stability.

#### Fundamental Properties of HE MXenes

Similar to HEMs, HE MXenes are composed of at least five principal elements in more or less equimolar proportions. The multi-principal elements increase the *S*_*conf*_ as well as the structural stability of the single phase. In addition, atoms with different radii cause severe lattice distortion, which improves the hardness and strength of HE MXenes. HE MXenes can achieve local chemical activity by regulating individual elements. The synergistic effects arising from multiple elements endow HE MXenes with unexpected and often unpredictable properties, addressing many of the stability and oxidation challenges encountered by single-transition-metal MXenes [207]. Otherwise, the 2D structure of HE MXenes has a higher surface area and more catalytically active sites (the most common number of elements in M sites of solid solution MXenes was two). HE MXenes can achieve a monolayer-like graphene, using the selective etching method. The properties of HE MXenes are affected by the surface functional groups. New functional groups can lead to new surface reactions, and the structure and properties of MXenes can be effectively controlled by controlling surface functional groups [207]. Similar to MXenes, HE MXenes has excellent photothermal conversion performance and has potential application prospects in the biomedical field. HE MXenes also exhibits highly efficient electromagnetic wave absorption performance and also has the advantage of being lightweight, as an electromagnetic wave absorber [69].

#### Applications of HE MXenes

The exceptional properties of HE MXenes have attracted significant attention in various fields, including energy storage, electrocatalysis, biomedicine, and electromagnetic applications. In 2021, Yang et al. [214] demonstrated that mechanical strain within HE MXenes can induce Li deposition on HE MXenes without the formation of dendrites. The HE MXene-Li anode symmetric battery can achieve prolonged cycling stability (up to 1200 h) in an ether-based electrolyte. Furthermore, the complete battery, assembled by coupling the HE MXene-Li anode with a LiFePO_4_ cathode, demonstrates excellent cycling performance. At a current rate of 0.5 C, the initial capacity is 170 mAh g^−1^. After 50 cycles, the capacity can still be maintained at 150 mAh g^−1^, significantly surpassing that of conventional MXenes. The atomic layers of HE MXenes exhibit substantial compressive and tensile strains, which facilitate rapid Li ion transfer.

In addition, HE MXenes exhibit significant potential as electrode materials for supercapacitors. Rosen et al. [213] prepared HE MXenes (Ti_1.1_V_0.7_Cr_*x*_Nb_1.0_Ta_0.6_C_3_T_*z*_) as a supercapacitor electrode, achieving volumetric and gravimetric capacitances of 688 F cm^−3^ and 490 F g^−1^, respectively. Subsequently, the HE MXenes films were utilized as electrode materials for Zn-ion hybrid supercapacitors (ZHSCs) and LIBs, achieving capacities of 77 and 126 mAh g^−1^, respectively, along with impressive long-term stability (10,000 cycles for ZHSCs and 1,000 cycles for LIBs). Additionally, single-layer HE MXenes (Ti_2_V_0.9_Cr_0.1_C_2_T_*x*_) exhibited remarkable gravimetric capacitance (553.27 F g^−1^) when used as electrodes for supercapacitors [213, 215]. The HE MXenes (Ti_3_C_2_(N_0.25_O_0.25_F_0.25_S_0.25_)_2_), as an anode material for LIBs, demonstrates high capacity and a low open circuit voltage of 385.38 mAh g^−1^ and 1 V, respectively. HE MXenes show substantial promise in ZHSCs and LIBs. The 2D structure of HE MXenes, coupled with excellent catalytic activity, has also attracted significant attention in the biomedical field. Shen et al. [216] demonstrated that HE MXenes can be used to treat drug-resistant bacterial infections and promote the healing of infected tissues with minuscule side effects. Furthermore, Wang et al. [69] found that HE MXenes not only offer the advantage of being lightweight but also efficiently absorb electromagnetic waves.

### Challenges and Prospects of HEMs

HEMs, especially HEAs and HEOs, offer distinct advantages over traditional alloys, oxides, and other compounds, particularly in the areas of mechanical performance and battery catalysis. The primary advantage of HEAs and HEOs stems from the so-called cocktail effect, whereby the material properties can be finely tuned by modifying the active and inactive components. In HEAs, the strong synergistic interactions among functional components play a pivotal role in enabling high electrocatalytic activity, especially for inert, noble metal-free catalysts involved in reactions such as HER, ORR, methanol oxidation, and carbon dioxide conversion. Similarly, HEOs have been widely employed as active electrode materials in batteries, benefiting from original structure’s stability during electrochemical cycling thanks to the high-entropy effect. For instance, in sandwich-layered HEO cathodes, entropy stability facilitates the formation and maintenance of O3-type structures. Additionally, HE MXenes show considerable promise for applications in both battery technologies and biomedicine. However, despite these promising features, significant challenges hinder practical implementation. Although HEAs demonstrate mechanical properties that surpass those of many existing alloys, the large-scale production remains difficult due to current preparation method limitations. Moreover, HEAs still exhibit a ductility gap when compared to elemental metals. Typically, HEAs require high-temperature processing to form single-phase solid solutions, which further complicates fabrication. The compositional space of HEAs is vast and largely unexplored, making it a formidable task to identify optimal element combinations. While research on HEAs has advanced significantly, especially in understanding the impact of defects on structural and functional properties, many opportunities remain to investigate a broader range of defect types and more complex chemical environments. Similarly, the defect chemistry of HEOs is still not well understood. Furthermore, the cycling performance of HEOs in battery applications currently falls short when compared with state-of-the-art battery materials, highlighting the need for continued development in this area. Research on HE MXenes for battery electrodes and sensors is still at an early stage. Therefore, there is an urgent need for further exploration and in-depth research focusing on the structural and functional design of HE MXenes.

The commercialization of HEMs in battery applications faces significant challenges, primarily due to high costs and difficulties in scaling up production. Notably, the expensive prices of multi-metal raw materials (e.g., Co, Ni) and energy-intensive, complex synthesis methods (e.g., high-temperature sintering and sol–gel processes), combined with purity control issues, result in costs that substantially exceed those of established materials like lithium iron phosphate. Furthermore, industrial-scale production struggles with achieving uniform elemental distribution, and the scarcity of specialized equipment further limits mass-production capabilities. Despite these challenges, the unique advantages of HEMs—such as exceptional environmental stability across a wide temperature range (− 60 to 150 °C) and synergistic effects from multiple elements—offer distinctive opportunities in high-end markets, including aerospace and solid-state batteries. Strategies such as low-Co/Co-free designs, optimization of large-scale manufacturing processes (e.g., dry electrode technology), and supportive policies are expected to reduce costs and facilitate gradual penetration into high-energy–density battery applications [77, 217, 218]. Nonetheless, achieving large-scale commercialization will require carefully balancing performance benefits with economic viability.

## Applications of HEMs in Batteries

Electrode materials are fundamental to the energy storage and release processes in batteries. Despite substantial advances in battery materials over recent decades, challenges such as chemical stability, ionic and electronic conductivity, reaction kinetics, and crystal phase stability persist. Consequently, the development of electrode materials capable of addressing these challenges has emerged as a critical research focus in electrochemical energy storage. Recent studies have extensively explored materials such as carbon, organic compounds, metallic heterostructures, silicon suboxides, and Bi-based hydrides as anode materials for batteries [219–224]. However, many of these materials continue to suffer from inherent limitations, including low electronic conductivity, poor cycling stability, and high solubility in electrolytes. In addition, although metallic lithium is often considered as the ideal anode material due to its high theoretical capacity, its practical application is hindered by critical issues such as dendritic lithium growth, which poses serious safety concerns. On the cathode side, a wide range of materials—such as Ni-rich, porous organic polymers, Ni-doped porous carbon, manganese oxide, Na_3_V_2_(PO_4_)_3_, Na_2_Fe(SO_4_)_2_, O3-type layered, and PBAs have been extensively explored for use in LIBs, LSBs, ZIBs and SIBs [225–232]. While battery performance has improved, many cathode materials still suffer from intrinsic limitations, such as poor electronic conductivity, sluggish ion diffusion kinetics, and low theoretical specific capacity. These challenges are particularly evident in LSBs, where the cathode is further constrained by the low conductivity of sulfur and its reaction products, severe volume changes during charge–discharge cycles, and the shuttle effect caused by the diffusion of soluble LiPSs [227]. To address these persistent limitations in cathode design, attention has also shifted toward alternative battery systems, such as ZIBs, which offer advantages in safety and cost. However, the development of cathode materials for ZIBs must prioritize Zn^2+^ intercalation strategies and investigate new materials better suited for Zn^2+^ intercalation to ensure more stable Zn^2+^ storage. Therefore, the development of advanced electrode materials remains a central focus in battery research. In this context, the incorporation of high-entropy mechanisms has recently emerged as an effective strategy to tackle the aforementioned challenges. By harnessing the synergistic effects of multi-elemental systems, high-entropy materials offer improved structural stability, enhanced electrochemical performance, and greater design flexibility. This section provides a comprehensive overview of recent advances in the application of high-entropy anode materials, cathode materials, and electrolytes in various metal-ion batteries.

**Fig. 4:**
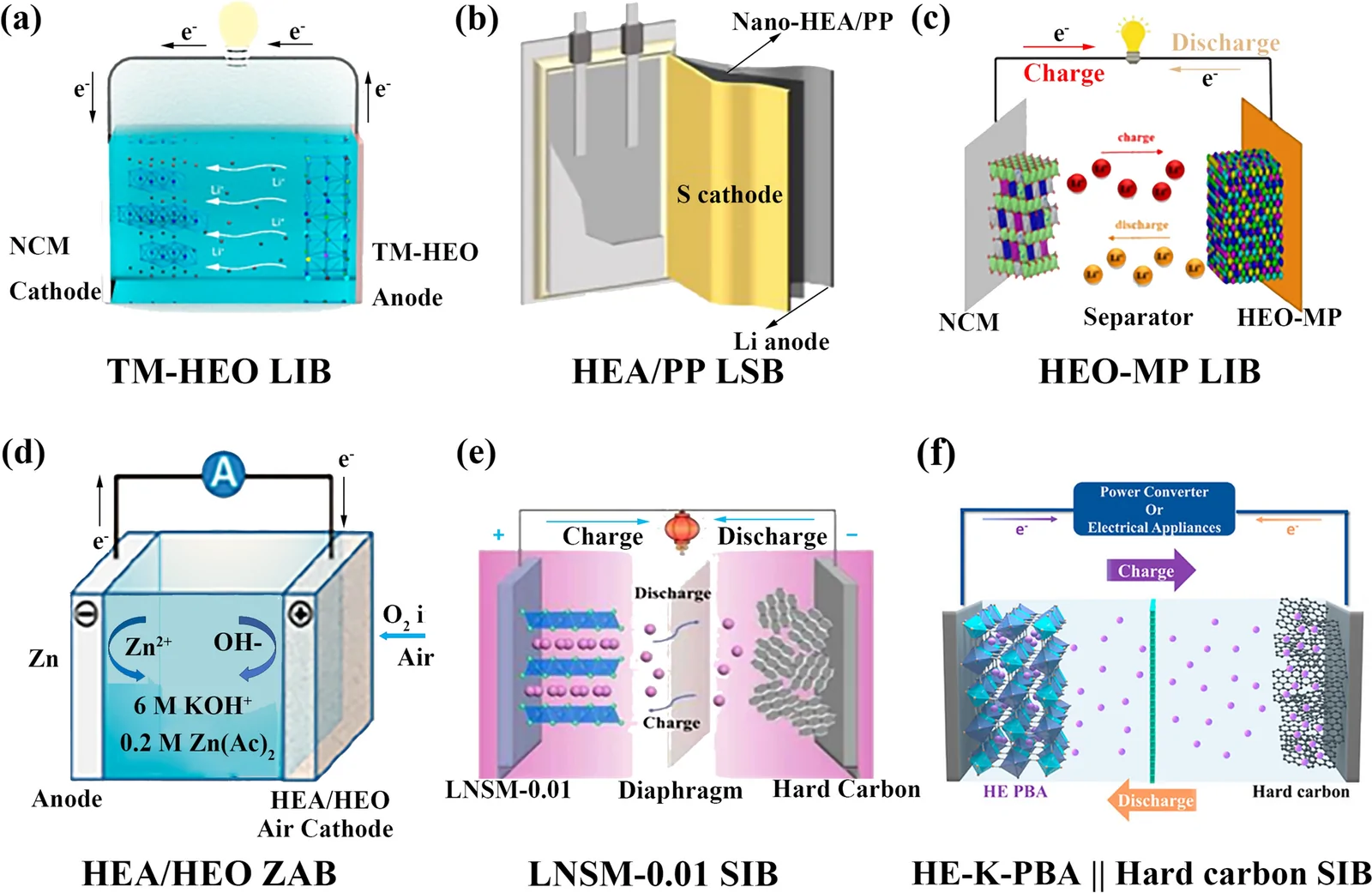
Schematic figure of **a** full-cell using NCM111 cathode and TM-HEO anode for LIB [187], Copyright 2019, Elsevier; **b** pouch cell of LSB [137], Copyright 2021, Elsevier; **c** full-cell of HEO-MP LIB [204], Copyright 2022, Elsevier; **d** HEA/HEO ZAB [240], Copyright 2020, American Chemical Society; **e** LNSM-0.01 SIB [241], Copyright 2023, Elsevier; **f** HE-K-PBA||Hard carbon SIB [202], Copyright 2023, American Chemical Society

HEMs, characterized by high-entropy effect, severe lattice dislocation, sluggish diffusion, and cocktail effects have drawn great interest for applications in batteries and energy storage. The compositional complexity of HEBMs underpins superior electrochemical performance, while also presenting significant challenges in material design. To address these challenges, designing HEBMs requires careful consideration of two key aspects: element selection and the chemical stoichiometry of components. Building on these design principles, the high-entropy concept has now been integrated into various materials, including TMOs, MXenes, spinel oxides, rock-salt oxides, PBAs, and O3/P3/P2 layered structures. Among these, transition-metal high-entropy oxides (TM-HEOs) exhibit excellent cycling stability and rate capability, making them promising candidates for use as anode materials in LIBs [146]. In addition, the spinel structure is well-known for facilitating effective Li^+^ transport. Specifically, oxygen vacancies, induced by the multiple valence states of cations in spinel HEOs, enhance Li^+^ conduction [195, 233]. Moreover, compared to rock-salt-structured HEOs, spinel-structured HEOs, with higher cation valence states and an additional Wyckoff site, facilitate the transfer of hypervalent ions. This additional site effectively extends the range of valence states during the charge–discharge cycle, thereby enhancing capacity. HESOs are promising electrode materials for next-generation LIBs. Notably, the lattice structure remains stable during cycling, resulting in smaller volume fluctuations compared to other TMOs. Such increased structural stability enhances long-term cycling performance by minimizing damage caused by volume expansion [72, 145, 159, 160, 234]. Furthermore, various HEBMs have been reported in the context of Li, Zn, Na, and other metal battery technologies [86, 91, 133, 140, 194, 196, 235–239]. HEBMs exhibit a broad spectrum of electrochemical performances that are intimately linked to diverse structural configurations. To systematically illustrate this relationship, Fig. 4 compares typical configurations across different battery systems, including (a) TM-HEO LIBs, (b) HEA/PP LSBs, (c) HEO-MP LIBs, (d) HEA-HEO ZABs, (e) LNSM-0.01 SIBs, and (f) HE PBA SIBs.

### HEMs in Lithium Batteries

LIBs have garnered increasing attention for applications in the growing electric vehicle and electronic device markets, owing to the superior charge storage performance, which includes long cycle life, high energy and power capabilities, and low maintenance costs [242]. Graphite was the initial commercial choice; however, its low theoretical capacity (372 mAh g^−1^) and unsafe charging profile at high current densities have driven the search for alternative anode materials [243]. Meanwhile, the performance of traditional LIBs is also constrained by electrolytes, which limit operation under harsh conditions. Therefore, developing electrolytes that can function effectively at low temperatures is equally crucial for advancing LIB technology. In this context, HEMs have emerged as promising candidates not only for anodes but also for cathodes and electrolytes, offering enhanced cycling stability, increased capacity, improved low-temperature performance, and cost benefits.

#### HEMs as Anode Materials for LIBs

With rapid societal advancement, the market share of electronic devices and electric vehicles is steadily increasing, driving significant innovation in LIBs. However, constrained by space limitations and volumetric weight, there is a pressing need for anode materials with higher energy densities to replace commercial graphite anodes. In comparison with conventional graphite anodes, TMO-based anodes can operate at higher potentials, thereby enhancing safety. Nonetheless, TMOs face several critical challenges, including maintaining cyclic stability and structural integrity. TM-HEOs exhibit excellent cycling stability and rate capabilities, making them promising candidates as anode materials for LIBs [155, 159, 244]. Due to the high *S*_*conf*_, the lattice structure of HEMs can be preserved during cycling, resulting in smaller volume fluctuations compared to other TMOs. Increased structural stability enhances long-term cycling performance by minimizing damage caused by volume expansion.

**Fig. 5:**
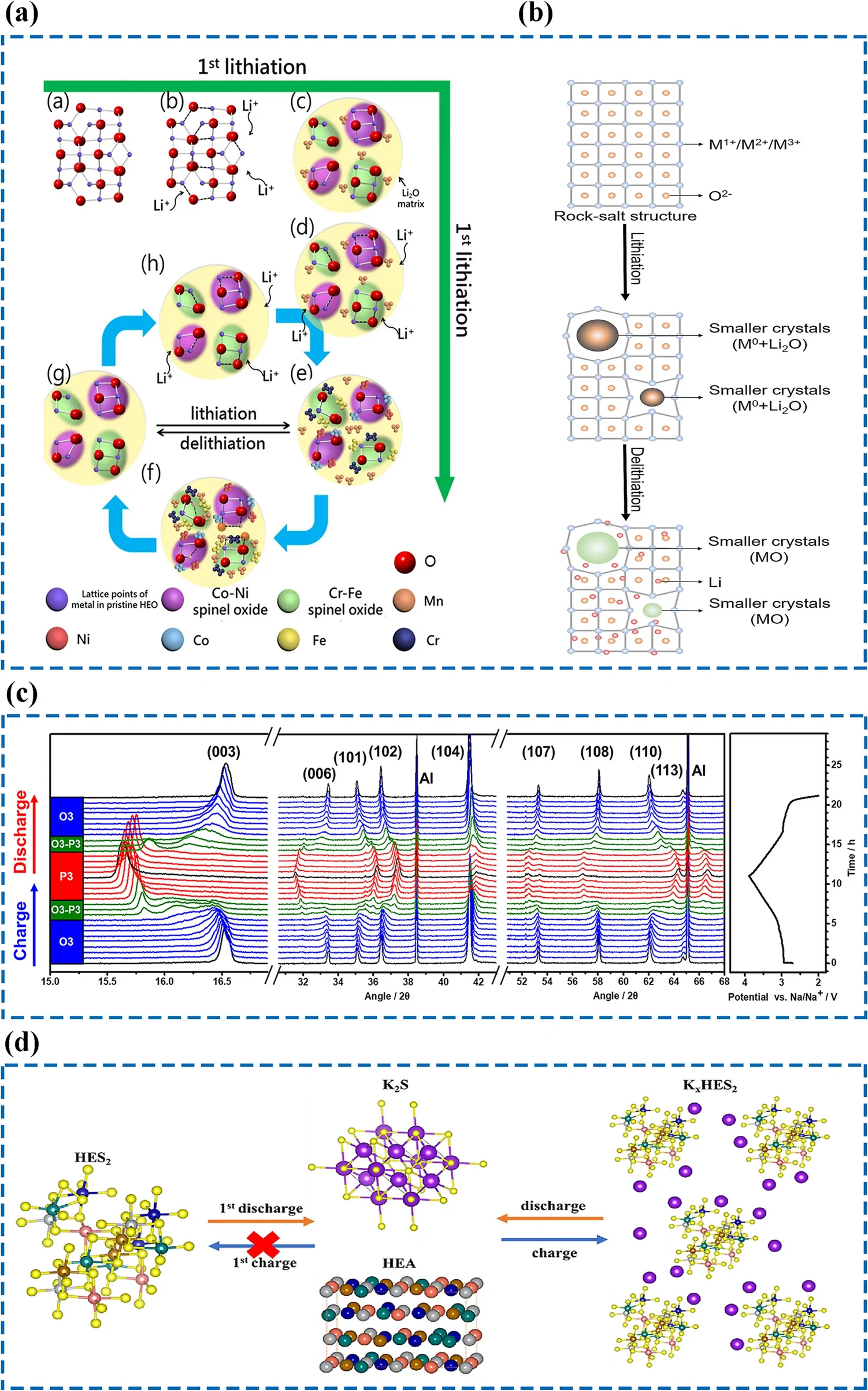
**a** Schematic illustration of atomic-scale microstructure evolution of (CrMnFeCoNi)_3_O_4_ HEO during lithiation/delithiation cycling [159], Copyright 2021, Elsevier.** b** Schematic diagram of the structural transition of the lithiation/de-lithiation of (MgCoNiCuZn)O HEO anodes for LIBs [247], Copyright 2023, Elsevier. **c** *Operando* XRD patterns and the corresponding voltage curve of the NaCu_0.1_Ni_0.3_Fe_0.2_Mn_0.2_Ti_0.2_O_2_ (NCNFMT) electrode during the first cycle at 0.1C, in the voltage range of 2.0–3.9 V vs. Na/Na^+^ [201], Copyright 2022, Elsevier. **d** Schematic illustration of the reaction mechanism of HES_2_ [248], Copyright 2023, Elsevier

HESOs and rock-salt HEOs have been extensively investigated as electrode materials for high-performance LIBs. HESO represents a promising electrode material for next-generation LIBs; therefore, a detailed atomic-level mechanistic study of its microstructural transitions during cycling is critical. Huang et al. [159] investigated the unique transformation behavior of HESO under various states of charge and cycle numbers. Atomic-scale structural evolution of HEO during lithiation/delithiation is shown in Fig. 5a. Although elemental segregation was observed in lithiated HEO particles, the spinel crystal structure remained intact, ensuring excellent cyclability. Zhai et al. [245] investigated the cycling performance of HESO as an anode material for LIBs over its entire lifecycle, categorizing the cycling process into three stages: activation, upgradation, and degradation. The porous HESO ((Cr_0.2_Fe_0.2_Co_0.2_Ni_0.2_Zn_0.2_)_3_O_4_) not only facilitates electrolyte transport but also mitigates volume changes in the active materials during cycling. Meanwhile, the rock-salt HEO (Mg_0.2_Co_0.2_Ni_0.2_Cu_0.2_Zn_0.2_O) demonstrates outstanding performance as a LIBs anode, delivering a high specific capacity of 490 mAh g^−1^ while maintaining exceptional cycling stability even under high current density [246]. Ren et al. [247] revealed that pristine (MgCoNiCuZn)O maintains a long-range ordered rock-salt phase. During lithiation, Li^+^ incorporation induces conversion reactions in TMs (Co^2+/3+^, Ni^2+/3+^, Cu^2+/1+^, Zn^2+^), while electrochemically inert Mg^2+^ plays a critical role in stabilizing the rock-salt matrix. Remarkably, upon delithiation, the reduced elements fully reoccupy the rock-salt lattice, achieving complete structural recovery (Fig. 5b).The entropy-induced stabilization effect suppresses the formation of cation short-range order within the crystalline structure of HEO through lattice distortion, thereby ensuring rapid Li⁺ transport and excellent electrochemical performance [157]. Furthermore, coating HEO with polyaniline (PANI) as an anode material for LIBs enhances the structural stability and electrical conductivity of the composite, while also suppressing the overgrowth of the solid electrolyte interphase (SEI) and the absorption of HF in the electrolyte through proton doping. The incorporation of multi-component HEO anodes can enhance both cycling stability and rate capability in LIBs. The newly created sites expand the range of valence states during the charge–discharge process, thereby improving capacity [204].

Combining high entropy with amorphization effectively addresses the issues of poor conductivity and mechanical instability in Si-based anodes, thereby enhancing cycle stability. The enhanced structural stability arises from the solid solution reaction mechanism involving Si, Al, Mg, and Ge, which mitigates phase separation [136]. However, traditional HESOs often rely on Co, a toxic, expensive, and scarce element. Therefore, substituting Co with cost-effective and environmentally benign elements has become a key research focus in alkali-ion batteries. In addition, the fabrication of interconnected 3D nanoporous structures reduces ion transfer distances and facilitates electron conduction, thereby improving overall electrochemical performance. In HESOs, Co can be substituted with V or Ti while maintaining high capacity. The as-prepared HESO exhibits the highest capacity, reaching 730.2 mAh g^−1^ at 2.0 A g^−1^. Nanoporous HEOs re self-activated during cycling, exhibiting a gradual increase in capacity following an initial decrease [249]. The double yolk-shell structure of HEO ((CrMnFeCoNi)_3_O_4_), designed by Zhang et al. [250] demonstrates exceptional performance as an anode material for LIBs, including reducing ion migration barriers, accommodating volume expansion, and alleviating stress. The diverse structures of HEMs effectively mitigate stress induced by Li^+^ intercalation, prevent collapse due to structural volume expansion, and withstand the rapid shuttle of Li^+^ [251, 252]. The key characteristics of LIBs utilizing HEMs as anode materials are summarized in Table 4.

**Table 4:** The compositions, structures, capacity, rate capability, and cycle performance of HEMs as anode material for LIBs

Type	Compositions	Structures	Capacity mAh g^−1^	Rate capability /mAh g^−1^	Cycle performance/mAh g^−1^ (cycles)	Refs
LIBs	Gd(Co_0.2_Cr_0.2_Fe_0.2_Mn_0.2_Ni_0.2_)O_3_	Perovskite	300	135 at 2 A g^−1^	394 (500) at 1 A g^−1^	[11]
	(CoMnZnNiMg)_2_CrO_4_	Spinel	1483.2	371 at 2000 mA g^−1^	608 (200) at 200 mA g^−1^	[162]
	(CrMnFeNiZn)O	Spinel	865	560 at 3000 mA g^−1^	90% (200) at 500 mA g^−1^	[164]
	K(MgMnFeCoNi)F_3_	Perovskite	1327	50 at 3.2 A g^−1^	120 (2000) at 2 A g^−1^	[172]
	(FeCoNiCrMn)_2_O_4_	Spinel	1645.3	482.5 at 3 A g^−1^	596.5 (1200) at 2 A g^−1^	[187]
	(FeCoCrMnZn)_3_O_4_	Spinel	514.6	302 at 3 A g^−1^	828.6 (1200) at 2 A g^−1^	[233]
	(MgCoNiCuZn)O	Rock-salt	822.7	325 at 10 A g^−1^	261 (3000) at 4 A g^−1^	[204]
	Zn_0.5_Co_0.5_Mn_0.5_Fe_0.5_Al_0.5_Mg_0.5_O_4_	Spinel	290	80 at 8 A g^−1^	223 (5000) at 2 A g^−1^	[252]
	(FeCoNiCuZn)_3_O_4_	Spinel	716.5	306.6 at 5 A g^−1^	540 (500) at 1 A g^−1^	[253]
	(CoNiZnFeMnLi)_3_O_4_	Spinel	1104.3	293 at 2000 mA g^−1^	605 (100) at 100 mA g^−1^	[254]
	(CoMnFeCuNi)O	Spinel	1549	791 at 3 A g^−1^	1252 (100) at 0.2 A g^−1^	[255]
	(MgCoNiCuZn)O	Rock-salt	525	150 at 10 A g^−1^	80% (200) at 1 A g^−1^	[247]
	(MgCoNiZn)_0.65_Li_0.35_O	Rock-salt	1930	925 at 100 mA g^−1^	610 (130) at 1000 mA g^−1^	[256]
	Ge-Sn-Sb-Si-Fe-Cu-P	Dragon-fruit	1448	787 at 2000 mA g^−1^	800 (1600 h) at 100 mA g^−1^	[257]
	(NiCoCuMgFe)_2_O_4_	Porous hollow	907	937.1 at 2 A g^−1^	1131 (200) at 0.1 A g^−1^	[258]
	(Co_0.2_Cr_0.2_Fe_0.2_Mn_0.2_Ni_0.2_)_3_O_4_	Spinel	1133	428 at 10 A g^−1^	980 (50) at 0.1 A g^−1^	[259]
	(VCrNiCoMn)_3_O_4_	Spinel	1269	394 at 2 A g^−1^	733 (1000) at 0.2 A g^−1^	[244]
	(CrMnFeCoNi)_3_O_4_	Yolk–shell	2435.15	1721 at 0.5 A g^−1^	1356 (500) at 1 A g^−1^	[250]

#### HEMs as Cathode Materials for LIBs

The cathode material in LIBs typically exhibits lower specific capacity and stability compared to intercalation anode materials, thereby becoming a critical factor that limits the overall performance of the battery. To address this limitation, HEO materials have also been introduced as cathode materials for LIBs. In particular, disordered rock-salt-type HEMs, characterized by remarkable chemical flexibility, demonstrate high capacity as cathodes for LIBs [83]. Beyond disordered rock-salt-type HEMs, layered structures have emerged as another promising configuration for high-entropy cathodes. In particular, Ni-containing layered TMOs have attracted considerable attention for high theoretical capacities and potential in commercial applications [260]. High-entropy layered oxides are generally considered to have greater potential, owing to the presence of 2D ion migration channels between layers. The surface phase of high-entropy layered oxide (LiNi_0.2_Mn_0.2_Co_0.2_Fe_0.2_Al_0.2_O_0.2_) effectively prevents the re-intercalation of Li^+^ during subsequent cycles [261].

To enhance battery safety, there is an urgent need for Co-free materials to be developed as cathodes for Li batteries. Here, high-entropy layered oxides, enriched with Ni and free of Co, exhibit exceptional thermal and cycling stability as cathode materials for LIBs [262]. During the initial delithiation process, high-entropy layered oxides undergo the formation of oxygen vacancies on the material surface and the reduction of metal ions, leading to the formation of a spinel-type TMO M_3_O_4_ (where M denotes a metal) surface phase. This surface phase impedes Li^+^ intercalation, resulting in rapid capacity degradation of the batteries. The introduction of F anions into (Li_1.2_Ni_0.15_Co_0.15_Al_0.1_Fe_0.15_Mn_0.25_O_1.7_F_0.3_) significantly enhances the initial discharge capacity and cycle stability of the battery. F ions inhibit the transition of the surface phase of Li^+^ high-entropy layered oxide materials from the layered to the spinel phase, thereby enhancing the structural stability of the material surface [263]. Additionally, several studies have employed high-entropy doping and coating strategies to enhance the structural stability and electrochemical performance of LIB cathodes [264–266].

#### HEMs as Electrolyte for LIBs

Electrolytes are a critical component in LIBs, facilitating ion conduction between the cathode and anode electrodes. In addition to enabling ion transport, electrolytes play a pivotal role in determining the reversibility and long-term stability of battery performance. To meet the demands of advanced LIB systems, suitable electrolytes must satisfy several key criteria: (1) exhibit high chemical and thermal stability to suppress side reactions; (2) provide high ionic conductivity while maintaining electrical insulation; (3) demonstrate adequate mechanical integrity, particularly in polymer-based systems; and (4) utilize cost-effective and environmentally friendly materials.

In comparison with conventional liquid electrolytes, the use of solid-state electrolytes (SSEs) can significantly enhance the energy density of batteries. However, the low ionic conductivity and restricted operating temperature of most SSEs limit applicability. Therefore, the development of novel SSEs with high ionic conductivity and improved interfacial contact is urgently required [193]. An increase in the *S*_*conf*_ allows for more free movement of ions and a quicker adaptation to changes in the electric field, thereby enhancing ion transport rates. A higher *S*_*conf*_ results in a more uniform charge distribution within the electrolyte. Nevertheless, there are limitations to *S*_*conf*_. When it becomes excessively high, the stability and durability of the electrolyte may be compromised [43]. HEMs exhibit superior structural stability, high ionic conductivity, and a large dielectric constant, which can effectively mitigate issues such as electrolyte precipitation and crystallization at low temperatures, as well as the formation of by-products during cycling, thereby improving battery capacity and lifespan.

A variety of high-entropy electrolytes (HEEs) have been developed, including non-aqueous liquid HEEs, aqueous liquid HEEs, high-entropy polymer electrolytes, and high-entropy inorganic electrolytes [43]. A rock-salt-structured Li-conducting HEO has been synthesized and utilized as an active filler in polyethylene oxide-based solid-state composite electrolytes. In galvanostatic plating/stripping tests, Li||Li symmetric batteries were cycled at a current density of 200 μA cm^−2^ for over 1200 h, with an overpotential of 140 mV. Additionally, a Li||LiFePO_4_ full cell was charged and discharged at 0.5 C for 100 cycles, maintaining a high capacity retention rate of 91%. These findings demonstrate the potential of Li-containing HEOs as fast ionic conductors for high-performance all-solid-state batteries. Garnet-type HEOs, known for excellent air stability, have also been investigated as air-stable solid electrolytes for LIBs [152]. HEO-Li exhibits a low ion migration barrier and strong interactions between HEO-Li oxygen vacancies and lithium salt anions, resulting in low voltage and extended cycle life [193]. HEEs enhance the performance of lithium metal batteries by increasing the diversity of electrolyte molecules. HEE-based LIBs can maintain high capacity and significant rate capability even at ultra-low temperatures [188]. Furthermore, the transport performance of Li^+^ and the fast-charging capabilities of batteries are improved when HEEs with smaller ion clusters are used [92]. In liquid HEEs, however, the excess entropy can promote crystallization, reducing the reversibility and lifespan of the electrolyte. One advantage of solvent-based HEEs is high ionic conductivity, such as 0.62 mS cm^−1^ [188]. By introducing local lattice distortions to limit the distribution of Cl^−^, the high-pressure limitations of halide solid electrolytes are addressed, effectively suppressing oxidation kinetics. Lattice distortion also strengthens Li–Cl bonds, facilitating favorable Li^+^ activation [267]. To enable a rigorous assessment of LIBs systems’ competitiveness, Table 5 systematically compares key electrochemical parameters including applications, compositions, structures, capacity, rate capability, and cycle performance. This multi-dimensional analysis provides critical insights for selecting optimal materials under specific operational requirements.

**Table 5:** The applications, compositions, structures, capacity, rate capacity, and cycle performance of HEM as cathode and electrolyte materials for LIBs

Type	Applications	Compositions	Structures	Capacity /mAh g^−1^	Rate capacity / mAh g^−1^	Cycle performance /mAh g^−1^ (cycles)	Refs
LIBs	Cathode	Mn-Co-Cr-Ti-Nb-F-O	Rock-salt	307	170 at 2000 mA g^−1^	76% (20) at 20 mA g^−1^	[83]
	Cathode	Mg-Co–Ni-Mn-Zn–O	Spinel-rock salt	2049.8	502.9 at 2 C	717.3 (1300) at 0.5 C	[268]
	Cathode	LiNi_0.9_Mn_0.05_Zr_0.01_Nb_0.01_Ti_0.01_Al_0.01_Mg_0.11_O_2_	Layered	212.81	206.35 at 0.1 C	76% (100) at 1 C	[217]
	Cathode	LiNi_0.2_Co_0.2_Al_0.2_Fe_0.2_Mn_0.2_O_2_	Layered	85.4	–	71.5% (100) at 0.1 C	[263]
	Cathode	Li_1.15_Na_0.05_Ni_0.19_Mn_0.56_Fe_0.02_Mg_0.02_Al_0.02_O_1.97_F_0.03_	Layered	221.3	123.7 at 0.5 C	76.8% (200) at 0.5 C	[269]
	Cathode	(La_0.2_Nd_0.2_Sm_0.2_Eu_0.2_Gd_0.2_)_2_Zr_2_O_4_	Coatings	190.5	194.7 at 0.1 C	74.2% (300) at 0.1 C	[265]
	Cathode	Li_0.9138_Co_0.9925_(La_0.0024_Al_0.0022_Mg_0.0039_Y_0.0029_)O_2_	Layered	147	93.9 at 20 C	72.5% (500) at 5 C	[266]
	Electrolyte	Li_6.4_La_3_Zr_0.4_Ta_0.4_Nb_0.4_Y_0.6_W_0.2_O_12_	Garnet	154	–	152 (5) at 0.1 C	[152]
	Electrolyte	HE EDF	Liquid	180	60% at 9 C	90% (1000) at 2 C	[235]

#### HEMs in LSBs

The reaction mechanism of LSBs differs from the deintercalation process in traditional LIBs. LSBs convert chemical energy into electrical energy through electrochemical reactions between Li and S. Due to the ultra-high theoretical specific capacity (1672 mAh g^−1^) of S, LSBs are considered one of the most promising post-LIBs technologies with high theoretical energy density up to 2600 Wh kg^−1^ [270, 271]. Furthermore, S is abundant, inexpensive, and non-toxic. However, the slow polysulfide conversion and the shuttle effect in LSBs hinder the practical development [272]. Therefore, a variety of methods have been employed to address the polysulfide shuttle effect, sluggish redox kinetics, and uncontrollable lithium dendrite growth in LSBs [273–275].

**Fig. 6:**
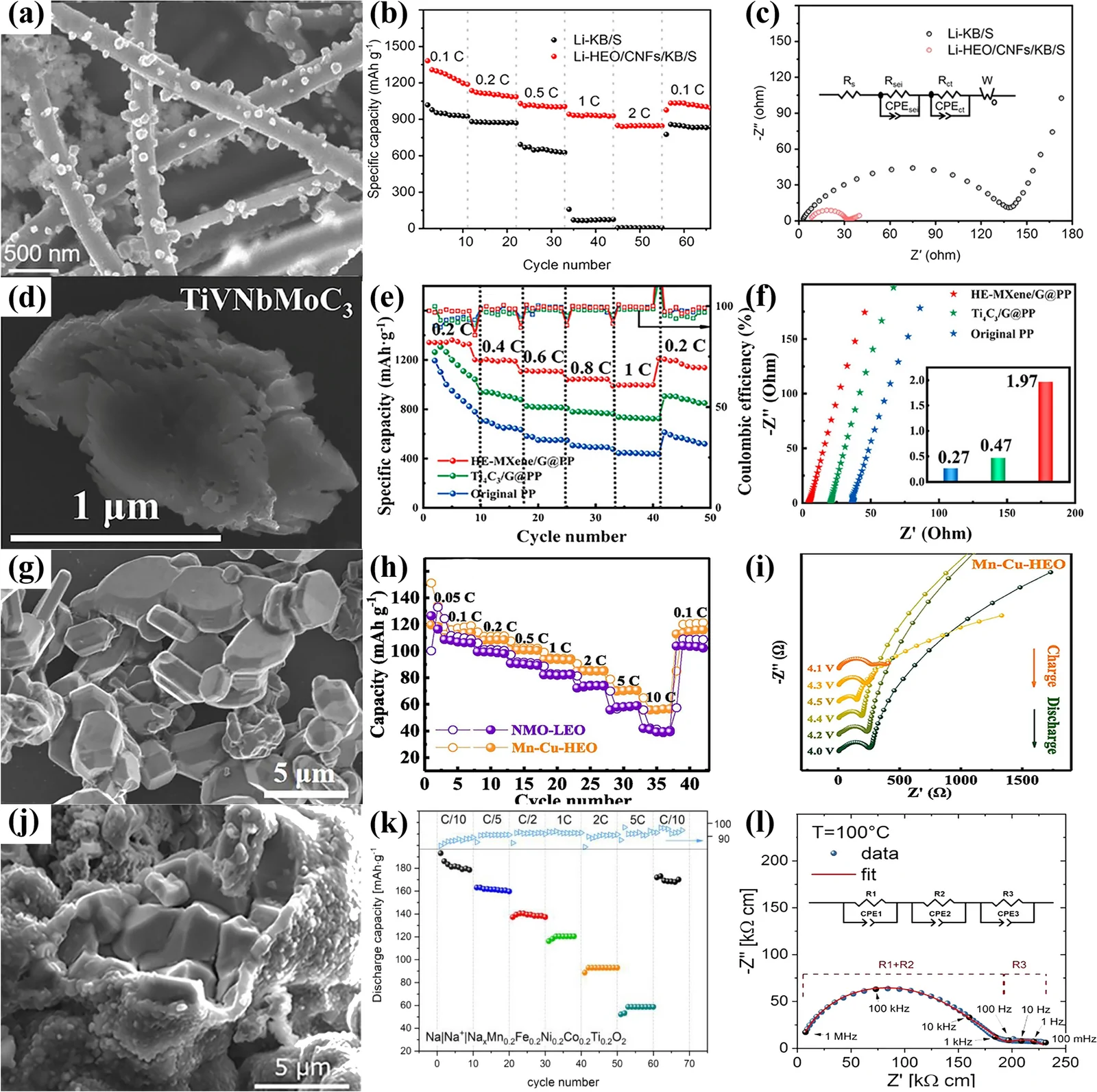
**a** SEM images of grapevine-like HEO composite, **b** Li-KB/S, and Li-HEO/CNFs/KB/S for LSBs, and **c** EIS plots with and without HEO/CNFs interlayers [276], Copyright 2024, Elsevier. **d** SEM images of TiVNbMoC_3_, **e** HE MXene/G@PP, Ti_4_C_3_/G@PP, and original PP for LSBs, and **f** EIS and inset illustrate the lithium-ion conductivity of the cells with pristine PP, Ti_4_C_3_/G@PP, and HE MXene/G@PP [277], Copyright 2024, American Chemical Society. **g** SEM images of high-entropy Mn-Cu-HEO, **h** Mn-Cu-HEO, and NMO-LEO for SIBs, and **i** Nyquist plots of Mn-Cu-HEO cathode [279], Copyright 2023, Elsevier.** j** SEM images of NaMn_0.2_Fe_0.2_Co_0.2_Ni_0.2_Ti_0.2_O_2_, **k** the first cycle of charging/discharging of NaMn_0.2_Fe_0.2_Co_0.2_Ni_0.2_Ti_0.2_O_2_, and **l** the frequency response of the sample measured at 100 °C presented as a Nyquist. Inset illustrates the equivalent circuit used for fitting the frequency response [280], Copyright 2022, Elsevier

The shuttle effect, which involves the dissolution and migration of LiPSs (Li_2_S_*m*_, 4 < *m* < 8) between electrodes during cycling, represents a major challenge causing active material loss and rapid capacity fading. HEAs address this issue through the inherent multi-element synergistic properties. The complex, entropy-stabilized surfaces of HEAs create numerous catalytically active sites that dramatically enhance polysulfide conversion kinetics while simultaneously providing strong chemisorption of polysulfide intermediates [85]. This dual functionality is achieved through several key characteristics: the optimized electronic structure of HEAs facilitates favorable interactions with sulfur species, the strained lattice generates defective sites that promote efficient nucleation of discharge products, and the high *S*_*conf*_ ensures sustained catalytic activity over extended cycling. These combined effects enable HEAs to maintain exceptional electrochemical performance, demonstrating both high polysulfide conversion efficiency and outstanding cycling stability compared to conventional materials. Specifically, the synergistic effect of Li–O and S–Ni bonds effectively alleviates the shuttle of LiPSs between the cathode and anode [144]. HEA nanocrystallites embedded in nitrogen-doped carbon not only exhibit high electrocatalytic activity toward the conversion of polysulfide species but also facilitate the solid-to-solid conversion from Li_2_S_2_ to Li_2_S. Furthermore, N-doped C, acting as a Lewis base substrate, enhances the ability to trap LiPSs. The synergy between HEA nanocrystallites with rapid ORR activity and N-doped C improves the cathode’s S utilization efficiency and specific capacity [85]. HEO nanofibers, containing multiple metal cations, offer abundant binding sites for the chemical entrapment of LiPSs and exhibit a significant synergistic effect in promoting the diffusion and conversion of LiPSs, as well as the deposition and dissolution of Li_2_S [191]. The SEM image in Fig. 6a reveals the distinctive grapevine-like architecture of the HEO/carbon nanofibers (HEO/CNFs) interlayer, which simultaneously facilitates rapid Li^+^ and e^−^ transport while physically constraining polysulfide migration. The unique HEO-NPs network actively engages with LiPSs through metal-sulfur bonding, effectively anchoring these active species. Electrochemical characterization (in Fig. 6b, c) demonstrates the Li-HEO/CNFs/KB/S composite’s superior performance compared to conventional Li-KB/S, delivering an initial capacity of 1381 mAh g^−1^ at 0.1 C with excellent cycling stability. The notably reduced charge transfer resistance observed in EIS measurements confirms this bifunctional interlayer design successfully addresses both polysulfide shuttling and reaction kinetics limitations in LSBs [276]. Additionally, HE MXenes and high-entropy phosphate oxide nanofibers have been explored for LSB applications. Figure 6d clearly demonstrates the successful fabrication of HE MXene-doped graphene composite (HE MXene/G) through chemical etching, where the Al layers were effectively removed via van der Waals forces while preserving the multi-layered nanostructure containing multiple elemental quasi-atoms. As evidenced in Fig. 6e, the PP separator modified with HE MXene/G (named HE MXene/G@PP) exhibits outstanding electrochemical performance, maintaining remarkable specific capacity even at high current rate of 1 C. Furthermore, the significantly reduced impedance in EIS measurements and superior lithium-ion conductivity (Fig. 6f) confirm the enhanced charge transfer kinetics enabled by this unique heterostructure design [277]. High-entropy phosphate oxide nanofibers efficiently catalyze the liquid-to-solid conversion process in LSBs, while HE MXenes, with high conductivity, facilitate rapid redox kinetics for LPS conversion. The abundant metal active sites on HE MXenes enable efficient chemical adsorption of LiPSs. Moreover, HE MXenes, exhibiting strong Li affinity, promote the uniform deposition of Li^+^ on the surface of Li metal. Furthermore, a catalyst composed of N-doped carbon, CNTs, and HEA-NPs significantly accelerates the bidirectional conversion of LiPSs. The N-doped carbon, owing to its strong sulfophilic activity, effectively adsorbs LiPSs and mitigates the shuttle effect, while the CNT-based conductive network facilitates efficient electron and ion transport [278].

HEMs could effectively enhance the electrochemical performance of LSBs, contributing to the suppression of LiPSs dissolution and the mitigation of the shuttle effect. This dual-role capability significantly enhances overall battery performance, particularly in terms of rate capability and cycling stability, as shown in Fig. 6a–f. Table 6 systematically summarizes the key electrochemical performance parameters of various LSB systems, including applications, compositions, structures, capacity, rate capability, and cycle performance. This comprehensive comparison facilitates a direct evaluation of the relative advantages and limitations of different HEM-based LSB configurations across multiple critical performance metrics.

**Table 6:** The applications, compositions, structures, capacity, rate capacity, and cycle performance of the HEMs for LSBs

Type	Applications	Compositions	Structures	Capacity /mAh g^−1^	Rate capacity / mAh g^−1^	Cycle performance /mAh g^−1^ (cycles)	Refs
LSBs	Anchor	(NiMgCuZnCo)O	Rock-salt	1191	634 at 1 C	479 (600) at 0.5 C	[144]
	Catalyst	La_0.8_Sr_0.2_(Cr_0.2_Mn_0.2_Fe_0.2_Co_0.2_Ni_0.2_)O_3_	Perovskite	1437.4	748.5 at 5 C	714.2 (500) at 1 C	[75]
	Catalyst	FeCoNiMnZn	FCC	816	680 at 5 C	680 (500) at 2 C	[137]
	Cathode	Fe_0.24_Co_0.26_Ni_0.10_Cu_0.15_Mn_0.25_	–	1079.5	440.5 at 5 C	740.1 (960) at 1 C	[85]
	Cathode	(Cu_0.7_Fe_0.6_Mn_0.4_Ni_0.6_Sn_0.5_)O_4_	Grapevine-like	1381	324 at 8 C	435 (400) at 1 C	[276]
	Cathode	(CrMnFeNiMg)_3_O_4_	Spinel	903	261 at C/5	560 (200) at C/20	[281]
	Cathode	(Co_0.2_Cu_0.2_Mg_0.2_Ni_0.2_Zn_0.2_)O	Rock-salt	1175	520 at 1 C	650 (250) at C/5	[282]
	Cathode	MgCrMnFeCoNi–O	Honeycomb	1396.9	857.5 at 3 C	1100 (1200) at 0.5 C	[283]
	Cathode	Li_0.5_(Zn_0.25_Mg_0.25_Co_0.25_Cu_0.2_^5^)_0.5_Fe_2_O_3.5_Cl_0.5_	Spinel	648	–	530 (100) at 0.1 C	[284]
	Bifunctional mediator	TiVNbMoC3/G@PP	MXene	1069.2	1001.5 at 1 C	509.36 (1200) at 2 C	[277]
	Dual-functional	Mn_0.4_Co_0.4_Ni_0.4_Cu_0.4_Zn_0.4_[Fe(CN)_6_]_2_	PBA	865.7	512.8 at 1 C	417.6 (850) at 1 C	[285]
	Electrolyte	Li_7_La_3_V_0.5_Ti_0.5_Al_0.5_Zr_0.512_	Perovskite	–	95 at 0.2 C	185 (6) at 0.1 C	[171]
	Electrocatalysts	CNT/HEA-NC	CNT	1109.3	521.1 at 5 C	692 (300) at 1 C	[278]
	Electrocatalysts	Pt_0.25_Cu_0.25_Fe_0.15_Ni_0.2_	Hollow	1077.9	338.2 at 5 C	768.5 (43) at 0.1 C	[117]

### HEMs in Zinc Batteries

Although LIBs are widely adopted for excellent performance, as discussed in Sect. 3.1, relatively limited energy densities restrict use in portable solid-state power conversion systems that demand higher energy output. In contrast, Zn-based rechargeable batteries (such as ZIBs and ZABs) have attracted increasing attention due to high theoretical energy density (1218 Wh kg^−1^ for ZABs), as well as low cost, environmental friendliness, and strong electrochemical performance [286]. This section reviews the research advancements of HEMs in ZIBs and ZABs.

#### HEMs in ZIBs

AZIBs, known for abundant material reserves, low cost, and high safety, have attracted growing attention in recent years [287]. Among the various cathode materials explored for AZIBs, Mn-based compounds stand out due to favorable cost, high operating voltage, and considerable specific capacity [288]. Recently, HEMs have emerged as promising candidates for aqueous ion batteries. The densely packed arrangement of metal atoms and strong synergistic interactions among multiple elements in HEMs contribute to improved electrochemical properties. In particular, HEOs exhibit a broadened d-band and reduced orbital degeneration compared to single-metal counterparts, enabling more efficient electron transfer and superior rate performance in AZIBs. Furthermore, the pronounced lattice strain fields in HEOs provide excellent tolerance against the electrostatic repulsion of high-charge-density Zn^2+^ ions, thereby enhancing structural integrity and cycling stability. A representative HEO cathode material, Fe_0.6_Mn_0.6_Co_0.6_Ni_0.6_Cu_0.6_O_4_ (FMCNC), exhibits outstanding electrochemical performance. This is primarily attributed to multi-element synergistic effects, which facilitate localized charge compensation in Co-HEOs and enhance electron/ion diffusion. These effects significantly improve reaction kinetics, resulting in one of the highest reported rate capacities (136.2 mAh g^−1^ at 10.0 A g^−1^) in AZIBs [194]. Additionally, HE MXene nanosheets, with excellent electronic and adsorption properties, are promising anode materials for ZIBs [210].

#### HEMs in ZABs

Rechargeable zinc-air batteries (RZABs) have emerged as a promising and sustainable energy storage technology due to high energy density, safety, and cost-effectiveness [289–291]. However, several critical challenges hinder widespread application, including: (1) passivation and dendrite formation on the zinc anode induced by alkaline electrolytes; (2) hydrogen evolution and corrosion of the zinc anode; (3) sluggish kinetics of the four-electron oxygen reactions (ORR and OER) at the air cathode; and (4) side reactions occurring on the surface of the air cathode [292]. Traditionally, precious metals and metal oxides, such as Pt, IrO_2_, and RuO_2_, have been regarded as efficient electrocatalysts for RZABs. Despite the high intrinsic catalytic activity, these materials suffer from several limitations that hinder practical application. Specifically, the sluggish kinetics of the OER and corrosion under charging conditions significantly compromise both activity and stability. In alkaline environments, the formation of intermediate species further accelerates catalyst degradation, while harsh electrochemical conditions often result in catalyst dissolution, ultimately leading to performance deterioration. Given these challenges, the development of OER catalysts that combine high catalytic efficiency with long-term stability is essential for advancing RZAB technology. As catalysts play a pivotal role in ensuring the reliable operation of these batteries, the search for alternative materials has intensified. In this context, HEAs and HEOs have emerged as highly promising electrocatalysts. The rich compositional diversity and lattice distortion in high-entropy catalysts enhance the d-band center of the material, modulating the binding strength of different reactants and lowering the activation energy of reactions. Moreover, the strong synergistic effects among multiple components are critical in improving the electrochemical stability of the material and facilitating efficient charge transfer. Compared to conventional systems employing Pt/C + IrO_2_-based, Ag + RuO_2_-based, Pt/C, and Pt/C + RuO_2_ catalysts, RZABs based on HEOs and HEAs catalysts exhibit significantly superior performance, including higher power densities, lower charge/discharge voltage differences, and longer cycling stabilities [133, 138, 240, 293–297].

Building upon the preceding discussion, HEAs and HEOs demonstrate superior electrochemical performance compared to traditional catalysts. A comprehensive summary of the electrocatalytic performance of HEAs and HEOs for ZABs is presented in Table 7. Owing to abundant active sites and robust chemical stability in alkaline environments, HEAs and HEOs significantly enhance the durability and power density of ZABs, thereby representing a promising alternative to conventional precious metal-based catalysts.

**Table 7:** The compositions, open circuit voltage, Tafel slope, power density, and cycle performance of HEMs as catalysts for ZABs

Type	Compositions	Open circuit voltage (V)	Tafel slope (mV dec^−1^)	Power density (mW cm^−2^)	Cycle performance	Refs
ZABs	FeNiCoMnRu@CNT	1.45	56	111	300 h at 10 mA cm^−2^	[133]
	Fe_6_Ni_20_Co_2_Mn_2_Cu_1.5_@rGO	1.405	48.9	154.612	40 h at 10 mA cm^−2^	[138]
	(AlNiCoFeCr)_3_O_4_	1.528	57	98.5	450 h at 2 mA cm^−2^	[293]
	Np-HEA/HEO	1.55	50	125	60 h at 2 mA cm^−2^	[294]
	(AlNiCoFeCr)_3_O_4_/Ag	1.528	49.3	146.4	150 h at 10 mA cm^−2^	[295]
	AlNiCoFeCrMnV-based oxide	1.50	54.6	162	700 h at 2 mA cm^−2^	[296]
	Al–Ni–Co-Ru-Mo nanowire	1.48	30.3	146.5	500 h at 2 mA cm^−2^	[240]
	AlNiCoRuMoCrFeTi	1.51	51.3	123.5	300 h at 10 mA cm^−2^	[297]
	FeCoNiCuMn	-	81	195	81 h	[298]
	Mn_70_Ni_7.5_Cu_7.5_Co_4.2_V_4.2_Fe_2_Mo_2_Pd_0.5_Pt_0.5_Au_0.5_Ru_0.5_Ir_0.5_	1.50	74.2	122	100 h at 400 mA cm^−2^	[299]
	MnNiCuCoVFeMoPdPtAuRuIr	1.486	86.7	279.4	300 h at 2 mA cm^−2^	[300]
	CrMnFeCoNi	1.489	37.9	116.5	16.6 days at 5 mA cm^−2^	[141]
	CC@FeCoNiMoRu-HEA/ C	1.48	55	89.9	180 h at 50 mA cm^−2^	[301]
	FeCoNiCuMo	–	42.4	298	788 h 10 mA cm^−2^	[302]
	FeCoNiMoW	1.59	36.7	116.9	660 h 8 mA cm^−2^	[303]

### HEMs in SIBs

SIBs hold significant promise for large-scale energy storage applications, owing to the low raw material costs, high energy density, improved safety, and stable performance across a broad temperature range [304]. However, the energy density of SIBs remains lower than that of commercial LIBs, primarily due to the limitations of low-capacity cathodes [197]. Over the past few decades, a variety of materials, including PBAs [305, 306], TMOs [307], polyanionic compounds [148, 308], and organic compounds [309, 310] have been extensively explored as cathodes for SIBs. While these materials exhibit distinct advantages, significant drawbacks remain. PBAs, in particular, are regarded as one of the most promising candidates for commercial SIB cathodes due to low cost, high theoretical capacity, 3D open framework, and ability to facilitate rapid Na^+^ transport [202]. However, PBAs are hindered by poor rate performance and substantial structural deformation during cycling, limiting overall effectiveness [178].

Layered TMOs, particularly O3-type layered oxides, exhibit high energy density and cost-effectiveness, making them widely adopted as cathode materials for SIBs. However, issues such as phase transitions, sluggish ion kinetics, and poor air stability during cycling limit application in high-performance solid-state SIBs [196, 199]. Polyanionic compounds (NaV_2_(PO_4_)_2_F_3_, NVPF) are notable for robust 3D grid structure and high thermal and electrochemical stability [147, 311]. Despite exhibiting high ionic conductivity and structural stability during Na^+^ extraction/insertion, these materials remain constrained by low electronic conductivity, high cost, low energy density, and poor rate performance [147–149, 182]. High-entropy cathode materials, particularly layered oxides, have attracted considerable attention for the potential to overcome key limitations, including poor structural stability, sluggish reaction kinetics, severe lattice distortion caused by the Jahn–Teller effect, and insufficient oxygen redox activity under high voltage [78, 312]. Although the incorporation of the high-entropy concept into PBA structures improves various performance metrics, severe particle agglomeration in HE PBAs hinders the fast-charging capability of SIBs. To mitigate this, Zhang et al. [313] proposed a hollow stepped spherical structure for HE PBAs, which not only reduces production costs but also effectively minimizes volume changes during cycling, thereby extending battery lifespan. Additionally, K-doped and single-crystal HE PBAs cathodes exhibit high reversible capacity and excellent rate performance [202]. O3-type compounds, which feature abundant oxygen vacancies, demonstrate ultra-long cycling stability and high capacity retention for SIBs [299]. By optimizing the O3/P2 ratio, high capacity and improved retention over a wide temperature range can be achieved [79]. Although O3-type layered oxides have been extensively studied as cathode materials for SIBs, the complex phase transitions and structural instability during sodium insertion/deinsertion remain significant challenges. To address these issues, incorporating multiple transition metals to construct high-entropy O3-type oxides can substantially enhance both structural and air stability, thereby effectively improving cycling performance in SIBs [314, 315].

To address environmental and health concerns, minimizing the use of toxic components in batteries is essential. As a result, Co-free high-entropy layered oxide materials have attracted increasing attention. In particular, high-entropy Co-free O3-type layered fluoride oxide materials have emerged as promising candidates for improving air stabilization in SIBs. The partial substitution of oxygen with fluoride effectively mitigates oxygen loss during prolonged cycling [77]. Furthermore, the O3-type high-entropy layered cathode materials with low Ni content and free Co exhibit a highly reversible O3–P3–O3 phase transition, occurring without strain accumulation after cycling. This characteristic significantly enhances long-term cycling stability. Within the high-entropy framework, Ni, Fe, and Cu ions act as charge compensators, while Mn, Zn, and Sn ions stabilize valence states and suppress interlayer sliding [198]. This well-balanced elemental synergy plays a crucial role in maintaining structural integrity. During the charge–discharge process, the layered oxide structure undergoes a reversible O3–P3 phase transition that remains stable even after prolonged cycling. This structural stability is primarily attributed to the entropy-driven stabilization effect, which effectively mitigates lattice distortion and phase degradation [82, 197, 316]. Liu et al. [317] proposed high-entropy P2-type layered oxides as cathode materials for SIBs, enhancing high-voltage capacity and cycle stability. For P2/O3 layered oxides, the high-entropy strategy not only optimizes the lattice structure and enhances structural stability during the Na intercalation/deintercalation, thereby suppressing undesirable phase transitions, but also facilitates ion transport kinetics and significantly boosts electrochemical performance [205, 318].

NASICON, a typical polyanionic compound, is frequently used as a Na^+^ cathode due to its 3D ion transport channels and abundant Na^+^ vacancies, which facilitate excellent Na^+^ diffusion kinetics. However, NASICON’s electronic conductivity and cycle stability during Na^+^ stripping/intercalation remain suboptimal. By applying the high-entropy effect to NASICON, structural stability is significantly enhanced, and the introduction of electrochemically active elements such as Ti, Fe, and Mn promotes multi-redox processes and alleviates structural degradation. This approach successfully achieves high energy density and long-term cycle stability [319]. The high-entropy effect further improves the thermal stability and conductivity of the electrode, accelerating charge transfer and diffusion dynamics during battery cycling [147, 149].

As cathode materials for SIBs, high-entropy layered oxides demonstrate superior electrochemical properties due to the unique structural design. As shown in Fig. 6g, the high *S*_*conf*_ and strong M–O bonding (M = Ti, Al, Zr, Y, La) synergistically stabilize the layered framework structure and local MnO_6_ octahedral environment, effectively suppressing phase transitions and volume changes during high-voltage cycling. This structural stability directly translates to superior electrochemical performance. Figure 6h, i demonstrates that Mn-Cu-HEO exhibits significantly superior performance than conventional low-entropy Na_0.65_Mn_0.84_O_2_ (NMO-LEO) materials across a wide rate range from 0.05 C to 10 C [279]. The high-entropy layered oxide also exists as large aggregates, as evidenced in Fig. 6j. Notably, the O3-P3 phase transition in NaMn_0.2_Fe_0.2_Co_0.2_Ni_0.2_Ti_0.2_O_2_ demonstrates partial reversibility during cycling, as shown in Fig. 6k. EIS analysis reveals sodium ions as the minority charge carrier (Fig. 6l) [280]. The structural evolution of high-entropy O3-type layered NaCu_0.1_Ni_0.3_Fe_0.2_Mn_0.2_Ti_0.2_O_2_ (NCNFMT) during the charging process is characterized in Fig. 5c. The NCNFMT material was identified as an O3-type layered structure prior to cycling. During the charging process, it undergoes an O3-P3 phase transition. At intermediate charging states, a subsequent O3 to P3 phase transformation occurs, accompanied by peak broadening and intensity reduction in the diffraction patterns due to the degradation of long-range ordering [201]. Table 8 composes critical metrics of SIBs using high-entropy cathodes, including energy density, capacity fade rates, and voltage decay, providing a comprehensive reference for material selection.

**Table 8:** The compositions, structures, rate performance, and capability retention rate, and Coulombic efficiency of HEMs as cathode materials for SIBs

Type	Compositions	Structures	Rate performance / mAh g^−1^	Capacity retention rate (cycles)	Coulombic efficiency	Refs
SIBs	Na(Fe_0.2_Ni_0.2_Ti_0.2_Sn_0.1_Li_0.1_)O_2_	O3	81 at 2 C	81%(100) at 0.5 C	98%	[76]
	NaNi_0.12_Cu_0.12_Mg_0.12_Fe_0.15_Co_0.15_Mn_0.1_Ti_0.1_Sn_0.1_Sb_0.04_O_2_	O3	87.1 at 5 C	83% (500) at 3 C	99.9%	[82]
	Na_2_(MnCoNiCuZn)_0.2_[Fe(CN)_6_]	PBA	52.3 at 20 C	83.1% (5000) at 10 C	99%	[177]
	NaNi_0.2_Fe_0.2_Mn_0.35_Cu_0.05_Zn_0.1_Sn_0.1_O_2_	Layered	64.3 at 2 C	87% (500) at 3 C	98%	[198]
	Na_0.85_Li_0.05_Ni_0.25_Cu_0.025_Mg_0.025_Fe_0.05_Al_0.05_Mn_0.05_Ti_0.05_O_2_	P2	81.8 at 10 C	~ 90% (1000) at 10 C	95.4%	[200]
	Na_3_V_1.9_(Ga,Mg,Al,Cr,Mn)_0.1_(PO_4_)_2_F_3_	Layered	71.4 at 50 C	80.4% (2000) at 20 C	~ 100%	[182]
	NaFe_0.2_Cu_0.1_Ni_0.2_Mn_0.3_Ti_0.2_O_2_	O3	70.2 at 5 C	83% (200) at 2 C	~ 99.9%	[203]
	Na_0.65_Cu_0.2_Li_0.06_Mg_0.15_Ti_0.015_Al_0.05_Zr_0.015_Y_0.015_La_0.015_O_2_	P2	55.5 at 10 C	87.2% (500) at 10 C	97.7%	[279]
	Na_0.7_Mn_0.4_Ni_0.3_Cu_0.1_Fe_0.1_Ti_0.1_O_1.95_F_0.1_	P2/O3	86.7 at 800 mA g^−1^	89.4% (200) at 50 mA g^−1^	97.6%	[79]
	Na_1.63_Mn_0.40_Co_0.22_Ni_0.06_Fe_0.06_[Fe(CN)_6_]_0.92_	3D	73 at 4 A g^−1^	80.1% (4000) at 2 A g^−1^	97.7%	[320]
	Na_0.79_Li_0.13_Ni_0.20_SS_0.01_Mn_0.66_O_2_	Layered	85.7 at 7 C	60 mAh g^−1 ^(1000) at 5 C	88.3%	[241]
	Na_0.9_Ni_0.2_Fe_0.2_Co_0.2_Mn_0.2_Ti_0.15_Cu_0.05_	O3	98.6 at 10 C	80.8 mAh g^−1 ^(2000) at 5 C	99.7%	[321]
	Na_*x*_(FeMnCoNiCu)[Fe(CN)_6_]_y□1-y_·n H_2_O	PBA	76.4 at 4 A g^−1^	75.6% (4000) at 1 A g^−1^	–	[313]
	NaNi_0.3_Cu_0.1_Fe_0.2_Mn_0.3_Ti_0.1_O_2_	O3	–	80% (682) at 1 C	91%	[314]
	NaNi_0.3_Cu_0.05_Fe_0.1_Mn_0.3_Ti_0.1_O_2_	O3	103 at 1 C	86.8% (500) at 5 C	70%	[315]
	Na_0.85_Li_0.05_Ni_0.3_Fe_0.1_Mn_0.3_Mg_0.05_Ti_0.2_O_2_	P2/O3	116 at 10 C	70% (100) at 0.5 C	97.6%	[318]
	Na_0.75_Cu_0.1_Fe_0.2_Mg_0.2_Mn_0.4_Ti_0.1_O_2_	P2/O3	165.32 at 0.2 C	86.73% (100) at 0.5 C	–	[205]
	Na_4_Fe_2.56_(NiCrMgCoMn)_0.027_(PO_4_)_2_P_2_O_7_	–	57 at 100 C	80% (4000) at 20 C	–	[322]
	Na(Fe_0.2_Co_0.15_Cu_0.05_Ni_0.2_Mn_0.2_Ti_0.2_)B_0.02_O_2_	O3	83 at 10 C	82% (300) at 10 C	90%	[323]

### HEMs in Potassium Batteries

The rapid advancement of renewable energy technologies aimed at replacing traditional fossil fuel has accelerated the demand for efficient electrical energy storage systems. Among emerging battery technologies, PIBs have attracted increasing attention due to favorable electrochemical characteristics. Specially, the low standard redox potential of K^+^/K is close to that of Li^+^/Li, enabling a high theoretical energy density (428.8 Wh kg^−1^) [324, 325]. Compared to SIBs (− 2.71 V versus standard electrode potential (*E*^*0*^)), PIBs (− 2.93 V* E*^*0*^) exhibit a higher operating voltage due to the more negative redox potential of potassium [325, 326]. Furthermore, unlike ZIBs (− 0.76 V *E*^*0*^), PIBs circumvent dendrite formation while delivering superior energy density, making them a promising candidate for next-generation energy storage systems [327]. PIBs are considered promising low-cost candidates for large-scale energy storage applications due to the abundant reserves of K resources [328]. However, to translate these advantages into practical performance, it is essential to develop suitable electrode materials that can accommodate the large ionic radius of K^+^ while maintaining structural stability and high capacity. In response to these challenges, various types of electrode materials have been explored. Depending on the K^+^ reaction behavior, electrode materials can be categorized into interaction compounds, carbon-based materials, alloy materials, and conversion materials [248]. In addition, due to the larger ionic radius of K^+^ compared to Li^+^, Na^+^, and Zn^2+^, significant structural stress occurs within the crystal lattice during charge–discharge cycles. Consequently, PIBs face challenges such as poor cycling stability and suboptimal rate performance [329]. Additionally, PIBs suffer from issues like drastic volume expansion of active materials and the shuttle effect of polychalcogenides. Therefore, there is an urgent need to discover new materials that offer superior ion transport properties and facilitate effective coupling between different components. 

Given the excellent performance of HEBMs in LIBs and ZIBs, application has now extended to PIBs, where outstanding rate performance and high reversible capacity are observed. Properly introducing local disorder through high-entropy doping in aqueous PIB cathode materials helps prevent phase transitions during charge and discharge cycles, thereby enhancing the material stability [330]. For example, high-entropy CoVMnFeZnPS_3_ (HEPS_3_) serve as an effective anode material by facilitating K^+^ ions transport and intercalation at the electrode interface, while simultaneously catalyzing the complete conversion of polysulfides and mitigating the shuttle effect. In addition, the uniformly distributed cations within its 2D structure induce a lattice distortion effect, which enhances mechanical stability. This distortion helps evenly distribution internal stress during K^+^ insertion and extraction, thereby preventing electrode pulverization and suppressing the aggregation of the MPCh_3_ layers [331]. NaCl-type high-entropy metal chalcogenides exhibit excellent electrochemical stability, even under high current densities, making promising candidates for PIBs. The superior performance can be attributed to the unique structural characteristics: active metals occupy cationic sites, which effectively lower the diffusion energy barrier for K^+^ ions and provide optimal adsorption energy to suppress the shuttle effect. Furthermore, the long-term cycling stability of PIBs is enhanced by the synergistic effects between nanoscale short-range structural units and the diverse functional metal nanoparticles generated during the energy storage process [332]. High-entropy metal disulfide (HES_2_), with the cluster structure, provide efficient transport paths for large-radius K^+^ ions, making more suitable for insertion/extraction. After potassiation, the resulting HEA exhibits a strong anchoring effect on polysulfides, significantly improving the capacity and cycling life of PIBs. The reaction formula is as follows (Fig. 5d): 

$${\mathrm{HES}}_{{2}} + x{\mathrm{K}}^{ + } + x{\mathrm{e}}^{ - } \to {\mathrm{HEA}} + {\mathrm{K}}_{{2}} {\mathrm{S}} \leftrightarrow {\mathrm{K}}_{x} {\mathrm{HES}}_{{2}}$$

During the initial potassiation process, HES_2_ reacted with K^+^ to form HEA + K_2_S_._ In the subsequent depotassiation process, HEA was converted to K_*x*_HES_2_ as the final product [248]. 

Recent breakthroughs in HEMs have unlocked unprecedented electrochemical performance in PIBs. Among them, HE PBAs stand out by delivering a highly reversible specific capacity and retaining retention of 84.4% after 3,448 cycles. This impressive stability can be attributed to robust crystal structure, which effectively resists dissolution, and solid solution reaction pathway, which undergo negligible volume change during charge and discharge. In addition, HE PBAs exhibit efficient ion transport kinetics, enabled by a reduced band gap and low energy barrier, further contributing to the excellent long-term performance [333]. High-entropy telluride (HET, Sb_1.4_Bi_0.2_Sn_0.2_Co_0.1_Mn_0.1_Te_3_) has emerged as a promising anode material for PIBs. The disordered coordination environment resulting from the high-entropy composition plays to crucial role in eliminating the bandgap, enhancing K^+^ adsorption, and lowering the migration barrier, thereby significantly improving the electrochemical kinetics. As a result, HET delivers a high initial specific capacity of 376.5 mAh g^−1^ at 50 mA g^−1^, along with an outstanding rate capability (175.7 mAh g^−1^ at 2,000 mA g^−1^), and excellent cycling stability with a service life of more than 500 cycles. In addition, K-ion full cell with a high energy density (428.8 Wh kg^−1^) was assembled [324]. Through the high-entropy charge compensation mechanism, the Mn^2+^ in the cathode has not oxidized to conventional Mn^3+^ during charging, but is in a valence state between + 2 and + 3, while the suppressed Jahn–Teller distortion leads to a solid solution reaction. In addition, theoretical calculations reveal the strong and insoluble structure of the electrode and the rapid diffusion of K^+^ ions in the crystal structure. With these advantages, the high-entropy cathode exhibits excellent cycling performance of more than 2000 cycles in dilute electrolyte. The full battery exhibits high energy density and long cycle life (> 1000 cycles), with a capacity retention rate of 88.1% at a current density of 0.5 A g^−1^ in dilute electrolyte, which has great potential for practical applications [334]. 

### HEMs for Wide-Temperature Batteries

The development of advanced battery systems capable of operating across extreme temperature ranges (− 50–80 °C) has become increasingly critical to meet the demands of modern applications such as electric vehicles operating in arctic conditions, aerospace missions in space’s thermal extremes, and polar research equipment requiring uninterrupted power in harsh environments [335]. Conventional battery technologies often suffer from severe performance degradation in these scenarios due to electrolyte freezing, sluggish ion kinetics at low temperatures, or accelerated interfacial side reactions at high temperatures, creating an urgent need for materials with exceptional temperature adaptability. In response to these challenges, HEMs have emerged as a groundbreaking solution, leveraging the unique entropy stabilization effects to overcome these limitations through multi-principal component design. These materials exhibit unparalleled advantages, including suppressed phase transitions, enhanced ion transport kinetics, and exceptional interfacial stability, enabling batteries to maintain high capacity retention (> 90%) even under extreme conditions. This section systematically examines cutting-edge research progress on high-entropy electrodes (anodes/cathodes) and electrolytes working under extreme temperatures, demonstrating the unprecedented performance in LIBs, LSBs, ZIBs, and SIBs systems. 

Among these advancements, the high-entropy stabilized SnSbMnBiTe alloy (HE-SSMBT) anode exhibits excellent low-temperature electrochemical performance for LIB. Its excellent conductivity, high tap density and ideal Young’s modulus together ensure the stable operation of the battery in extreme environments. The results shown that HE-SSMBT based LIBs can provide a specific capacity of up to 2299 mAh cm^−3^ at 0 °C; when the temperature drops to − 20 °C, it can still maintain a reversible capacity of 1838.3 mAh cm^−3^; even under harsh conditions of−30 °C, the initial specific capacity can still reach 1418 mAh cm^−3^, showing significant temperature adaptability [336]. Beyond anodes, the high-entropy stabilization strategy also proves highly effective in addressing the limitations of conventional cathode materials. A notable example is LiCoO_2_, which typically suffers from sluggish electrochemical kinetics at low temperatures and severe structural degradation under high-temperature or high-voltage conditions. To overcome these issues, Ren et al. [337] innovatively employed a high-entropy doping strategy to successfully enhance the performance of LiCoO_2_ across a wide temperature range (− 30 to 50 °C). The multi-component doping strategy in high-entropy LiCoO_2_ (HE-LCO) simultaneously addresses several critical issues: eliminating order–disorder phase transitions, alleviating lattice strain, enhancing lithium-ion transport kinetics, and suppressing electrochemical polarization. More significantly, the high-entropy modification dramatically reduces electrode/electrolyte interfacial side reactions, substantially improving interfacial stability. HE-LCO exhibits remarkable low-temperature performance, maintaining discharge capacities of 206.3, 193.2, 166.4, and 133.8 mAh g^−1^ at 0, − 10, − 20, and − 30 °C, respectively, demonstrating excellent capacity retention under extreme cold conditions. Additionally, the cycling performance of HE-LCO at 50 °C is significantly enhanced, with a capacity retention rate of 87.3% after 100 cycles at 1 C. The success of high-entropy electrodes like HE-SSMBT and HE-LCO in maintaining exceptional electrochemical performance under extreme temperatures highlights the transformative potential of entropy engineering in electrode design. However, realizing stable battery operation in such harsh environments requires more than just robust electrodes—electrolyte compatibility becomes equally critical. This necessity has driven increasing attention toward HEEs, which serve as essential counterparts to high-entropy electrodes for extreme-temperature applications. Zhang et al. [188] developed a decimal solvent-based HEE that achieves an ultra-low freezing point of − 130 °C (vs. − 30 °C for conventional electrolytes) through entropy stabilization, closely mirroring the stabilization mechanisms in high-entropy electrodes. This molecularly disordered system maintains 0.62 mS cm^−1^ conductivity at − 60 °C and enables full cell operation down to − 60 °C with 80% capacity retention at−40 °C, completing the wide-temperature battery system through unified entropy engineering principles. The advantages brought by the high-entropy effect extend to LSBs, which often suffer performance degradation under complex temperature environments. To tackle this challenge, Zhang et al. [338] innovatively employed a high-entropy enhanced dipole moment strategy by introducing multiple TM ions into LaSrMnO_3_, synthesizing HEO ultra-thin nanosheets La_0.71_Sr_0.29_ (Fe_0.19_Co_0.20_Ni_0.20_Zn_0.19_Mn_0.22_)O_3-*δ*_ (HE-LSMO). This design significantly enhances crystal asymmetry and dipole moments, improving LiPSs adsorption and conversion across − 35 to 50 °C. The sulfur/HE-LSMO (S/HE-LSMO) cathode delivers high initial specific capacity of 1,455.9 mAh g^−1^ at 50 °C (0.5 C) with a 71.1% retention after 100 cycles. Even at low temperature of − 35 °C, S/HE-LSMO can still maintain an initial capacity of 740.7 mAh g^−1^, and the capacity retention rate after 100 cycles is as high as 90.4%. 

In addition, the practical application of AZIBs is limited by the high liquid-to-solid transition temperature of aqueous electrolytes, so there is an urgent need to develop electrolytes that can work at low temperatures. Tian et al. [339] developed a HEE containing multi-component perchlorates (Zn, Ca, Mg, Li)ClO_4_. This HEE has an extremely low liquid-glass transition temperature (− 114 °C). The carbon composite material with iodine HEE Zn (CCM-I_2_|HEE|Zn) battery achieved a cycling capacity of up to 182 mAh g^−1^ at a current density of 100 mA g^−1^ and excellent cycling performance (the capacity was 102 mAh g^−1^ after 5,000 cycles at a current density of 5.0 A g^−1^). In addition, the study confirmed that the HEE exhibited excellent electrochemical performance at − 70 °C. Fan et al. [340] also constructed a HEE (Li_2_ZnBr_4_·9H_2_O) for ZIBs. In HEE, the unique properties of bromide ions exclude water molecules from the solvation structure, and the hydrogen bond network is destroyed by partially hydrated Li-Br, which significantly improves the low-temperature stability and ionic conductivity of the HEE. Therefore, the HEE-based Zn||Cu battery exhibits an ultra-long cycle life at 30 °C. Even when the temperature drops to − 30 °C, the Zn battery can still achieve a capacity retention rate of up to 71.2% after 800 cycles. 

Likewise, the remarkable temperature adaptability of high-entropy cathodes is further exemplified in SIBs. Zhou et al. [79] synthesized a high-entropy P2/O3 biphasic Na_0.7_Mn_0.4_Ni_0.3_Cu_0.1_Fe_0.1_Ti_0.1_O_1.95_F_0.1_ cathode that demonstrates outstanding capacity retention across − 40 to 50 °C, maintaining 99.5%, 94.5%, and 71.1%, respectively, with initial discharge capacities of 86.7, 91.2, and 128.4 mAh g^−1^, respectively. This performance originates from the synergistic combination of expanded interlayer spacing facilitating Na^+^ migration and high-entropy stabilization suppressing both the Jahn–Teller and transition metal layer slippage. Complementing this work, Dang et al. [323] prepared boron-doped O3-type HEO Na(Fe_0.2_Co_0.15_Cu_0.05_Ni_0.2_Mn_0.2_Ti_0.2_)B_0.02_O_2_ (NFCCNMT-B_0.02_), where the covalent B-O bonds and high *S*_conf_ ensured structural stability. The material delivers exceptional cycling performance (1 C/100 cycles, capacity retention 95%; 10 C/300 cycles, capacity retention 82%) and excellent rate capability (at 10 C, 83 mAh g^−1^), while maintaining high capacities across−20 to 60 °C (113.4 mAh g^−1^@−20 °C, 121 mAh g^−1^@25 °C, and 119 mAh g^−1^@60 °C, respectively). Further extending the operational limits, Du et al. [341] developed a high-entropy Na_3.45_V_0.4_Fe_0.4_Ti_0.4_Mn_0.45_Cr_0.35_(PO_4_)_3_ (HE-Na_3.45_TMP) as cathode for SIBs, demonstrating exceptional wide-temperature adaptability with stable operation across − 50 to 60 °C. Among, HE-Na_3.45_TMP cathode maintains an outstanding capacity retention of 92.8% after 400 cycles at − 40 °C, while delivering a considerable capacity of 73.7 mAh g^−1^ even under extreme low-temperature conditions (− 50 °C). This performance originates from the material’s unique ability to suppress transition metal ion dissolution. 

The collective findings underscore that high-entropy engineering represents a paradigm-shifting approach for developing temperature-resilient battery systems. By precisely controlling *S*_*conf*_ through multi-principal component design, these materials simultaneously achieve: (1) suppressed phase transitions and structural degradation, (2) enhanced ion transport kinetics across wide temperature windows, and (3) exceptional interfacial stability against electrochemical/mechanical stresses. The demonstrated performance metrics, which include more than 90% capacity retention at − 50 °C and stable operation up to 80 °C, establish HEMs as essential enablers for energy storage in aerospace, polar research, and electric vehicles under extreme environmental conditions. 

## ML for HEMs

The unique advantages of HEMs have attracted widespread attention in many fields, especially batteries. However, the multi-element composition of HEMs also brings huge uncertainties. Yeh calculated the number of alloy systems containing five to thirteen elements, which totals 7099 [23]. This number further increases when considering non-equimolar compositions and the additional of minor elements to tailor properties. Beyond this, metastable HEA designed through specialized strategies, polymorphism-induced HEA composites, and alloys incorporating immiscible HE-NPs at varying concentrations add even greater complexity and diversity. Given the vastness of the compositional space, traditional theoretical approaches such as calculation of phase diagrams (CALPHAD) and DFT face critical limitations. Although these methods have been successfully applied to smaller subsets of HEA compositions, CALPHAD only provides equilibrium phase diagram, limiting its applicability for metastable or disordered phase at higher temperature. Meanwhile, DFT calculations are computationally expensive and thus impractical for exhaustive exploration of large composition spaces. To overcome these challenges, a materials design strategy combining ML surrogate models with experimental design algorithms has emerged as a promising approach. This integrated framework accelerates the discovery and optimization of novel HEAs by efficiently navigating the complex, high-dimensional composition landscape [342]. 

ML accelerates the screening and optimization of HEA catalysts through data-driven approaches. Initially, ML integrates both experimental and computational data (such as DFT-calculated adsorption energies and d-band centers) to construct comprehensive databases encompassing compositional features (element types, concentrations, electronegativity) and structural characteristics (crystal types, local coordination environments). Supervised learning methods (e.g., random forests, neural networks) are then employed to predict catalytic activity, while unsupervised learning techniques (e.g., clustering) help explore novel compositional spaces. Chen et al. [343] designed a FeCoNiCuMo HEA system with high catalytic activity for CO_2_ reduction based on DFT calculations. By considering 1280 adsorption sites to predict adsorption energies, three independent ML models were developed, achieving high-precision predictions for the CO_2_ reduction reaction. This approach combined computational chemistry with data-driven modeling to efficiently screen and optimize the catalytic performance of the multi-component alloy system. HEAs have demonstrated substantial potential in the field of catalysis, but identifying HEAs that combine high activity, low cost, and entropy stability remains a challenge due to the vast number of possible alloys. Therefore, Xu et al. [344] developed a data-efficient, multi-objective Bayesian optimization framework that can effectively identify promising ORR catalysts. ML can be leveraged to predict the hardness of solid solution HEAs and verify mechanical properties. By optimizing multiple performance metrics simultaneously, ML can aid in designing HEAs that meet specific requirements [17]. ML has also been applied to predict trends in ductility and yield strength for RHEAs [345, 346]. Currently, ML is increasingly being utilized in the search for new high-entropy systems [347–349]. 

**Fig. 7:**
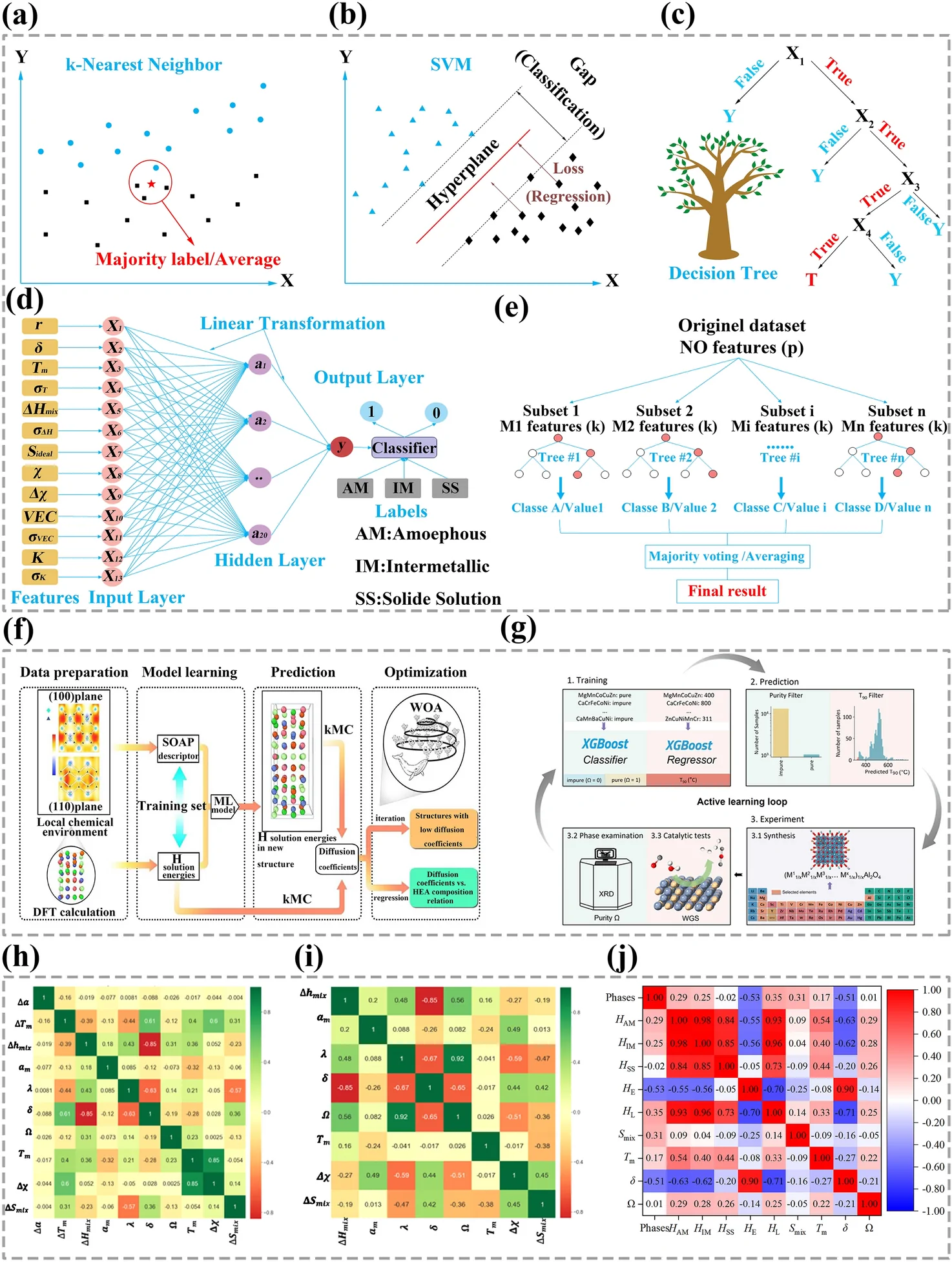
Schematic diagrams of **a** k-Nearest Neighbor, **b** SVM, **c** decision trees, **d** artificial neural network, and **e** random forest. **f** Graphic representation of the design strategy. Data preparation, model training, prediction, and optimization process for designing FeCoNiCrMn HEA with low H diffusion coefficients [351], Copyright 2021, Elsevier. **g** Training process of AL [353], Copyright 2024, American Chemical Society. Heat map displaying the correlation values between the features of **h** phases dataset and **i** Young’s modulus (E) dataset, as employed for the different multi-component alloys. A value close to -1 or 1 implies a negative or positive correlation, respectively [354], Copyright 2020, Elsevier. **j** Pearson correlation coefficients with the nine thermodynamics variables [355], Copyright 2020, Elsevier

Various ML algorithms are available, including linear algorithms, decision trees, logistic regression, support vector machines (SVM), random forests, gradient boosting classifiers, and artificial neural networks (ANN), as shown in Fig. 7a–e [350]. In HEA research, both traditional methods and deep neural networks are employed. For building robust ML models, comprehensive data collection, feature engineering, and careful model training and validation through cross-validation are crucial. For instance, Rao et al. [19] combined ML with DFT, thermodynamic calculations, and experiments to identify two high-entropy Invar alloys from millions of possible compositions. Zhou et al. [351] utilized the whale optimization algorithm to establish a quantitative relationship between the low hydrogen diffusion coefficient and HEA element composition (Fig. 7f). ML can also predict the solid-solution strength and hardness of HEAs, with an accuracy of 87% in predicting HEA structures [352]. This approach links element features with metastable states, thereby accelerating the discovery of potential components. Using the k-Nearest Neighbor algorithm to predict both quaternary and quinary high-entropy diborides, testing accuracy reached ~ 90% [347]. Additionally, using an optimization classification model, the accuracy for identifying solid-solution and non-solid-solution HEAs was 88.7%, and the accuracy for distinguishing between BCC, FCC, and dual-phase HEAs reached 91.3%.

Predicting crystal structures is crucial to overcoming the reliance on chance, thus promoting the discovery and design of high-performance noble oxides. In this context, recent studies have leveraged machine learning to facilitate the structural and functional prediction of HEOs. Liu et al. [356] developed three ML models to predict the crystal structures of HEOs, revealing that the ratio of anion to cation radius has the greatest impact on the crystal structure. Expanding beyond structural prediction, Mehrabi-Kalajahi et al. [357] applied ML models, including adaptive boosting, classification boosting, random forest, and extreme gradient boosting, to predict the selectivity of oxidation reactions in noble metal-free HEO reduced graphene oxide (HEO-rGO) nanocomposites. Among these, adaptive boosting achieved an error of less than 2.5%, and the predicted HEO composites demonstrated excellent catalytic performance. 

Due to the vast combinatorial space of potential elements, designing HEOs with ideal properties in a high-dimensional synthetic space has traditionally relied heavily on expert knowledge and intuition. ML, however, can help identify HEOs with unique properties by effectively leveraging limited experimental data. For instance, Nie et al. proposed an active learning (AL) strategy (Fig. 7g), which involved multiple iterations of AL to identify novel HEOs from a large pool of potential compositions. These HEOs exhibited excellent stability and outstanding H_2_ evolution rates [353]. Similarly, Zhang et al. [358] used a hybrid, knowledge-assisted, data-driven ML strategy to discover A_2_B_2_O_7_-type HEOs with low thermal conductivity (*k*). The predicted key physical parameters (KPP) related to the thermal conductivity of HEOs were highly consistent with experimental results. Furthermore, cross-validation of various models revealed the coupled effects of lattice vibration and charge on heat transfer, indicating that lattice distortions, identified by weak bonds and cation radius ratios with low electronegativity and small bonded charge density, can effectively reduce thermal conductivity. These advances underscore the potential of ML-driven methods to accelerate the discovery of high-performance, multi-functional HEOs across different physical regimes. However, despite such progress, certain key mechanical properties remain poorly understood. In particular, the fracture toughness of HEOs is critical for structural and thermal applications, yet quantifying the toughening mechanisms induced by lattice engineering and deformation remains a significant challenge. 

The core of ML lies in statistics, and it relies heavily on the correct selection of material features as data inputs. For high-entropy composites, essential structural and thermal features include parameters such as *δ*, *Δχ*_*Pauling*_, *ΔH*_*mix*_, *ΔS*_*mix*_, *Ω*, *T*_*m*_, and *VEC*. Figure 7h–j illustrates the correlation between the properties of HEMs, providing valuable insights for constructing more effective HEM models [354, 355]. Although ML still faces significant challenges in accurately predicting the performance of HEBMs due to issues such as small sample sizes, complex structures, element diversity, and high dimensionality, ML technology holds immense potential in discovering new materials, optimizing chemical processes, and predicting battery lifespan.

The choice of ML model has a decisive influence on the accuracy and computational efficiency of prediction results. To improve prediction accuracy, researchers often use strategies such as multi-model iterative optimization or multi-objective Bayesian framework [343, 344]. A major challenge lies in the stringent requirements for training data: ML models demand large, high-quality datasets with consistent synthesis conditions and characterization methods to ensure reliability. However, experimental data from different research groups or literature sources often vary in sample preparation protocols, measurement techniques, and reporting standards, making cross-study predictions highly unreliable. Additionally, the iterative process of material synthesis guided by ML predictions is resource-intensive, as each new composition requires experimental verification to confirm its properties, which may not align with theoretical predictions due to unforeseen kinetic or thermodynamic barriers during synthesis. Furthermore, the lack of standardized databases for HEMs exacerbates these issues, as inconsistent data formats and missing metadata hinder model generalization.

## Summary and Perspective

This comprehensive review explores recent advancements in HEMs for energy storage applications. It begins with a historical overview and conceptual framework, highlighting how the strategic incorporation of multiple metallic species in alloys as well as additional cations in oxides, significantly increases the S_*conf*_ of these systems. This entropy-driven design paradigm facilitates the formation of thermodynamically stable single-phase solid solutions with exceptional structural integrity, which in turn underpins their superior electrochemical performance. Beyond structural stability, the unique features of HEMs—including pronounced lattice distortion, sluggish diffusion kinetics, and the so-called cocktail effect that synergistically enhance the mechanical robustness and electrochemical performance. These remarkable properties have established HEMs as transformative materials across various high-technology sectors, particularly defense, aerospace engineering, and advanced energy storage systems. Our discussion places particular emphasis on recent breakthroughs in the integration of HEMs within contemporary battery technologies, including LIBs, LSBs, ZABs, ZIBs, SIBs, and PIBs.

The deliberate incorporation of multiple elements with diverse atomic radii in HEMs induces substantial lattice strain and defect formation, significantly enhancing mechanical hardness through solid solution strengthening. Under conditions of high S_*conf*_, these systems preferentially form single-phase solid solutions with exceptional thermodynamic stability. Extensive research has focused on the elemental composition, synthesis methods, structural characteristics, and characterization techniques of HEAs. Considerable progress has been made, with state-of-the-art methodologies enabling the fabrication of HEAs containing more than ten distinct metallic elements. Moreover, advanced nanofabrication techniques have facilitated the controlled synthesis of various two-dimensional nanostructures, including nanosheets, nanotubes, and nanoparticles, via diverse preparation routes. A defining feature of HEMs is inherently high defect concentration, particularly oxygen vacancies, which enhance Li^+^ ion intercalation and diffusion kinetics. This effect arises from the synergistic interplay of variable atomic radii and multi-valent cation states within the material matrix. Notably, certain cations (e.g., Mg^2+^) function primarily as structural stabilizers rather than redox-active species, thereby improving cyclability without compromising capacity. The successful synthesis of HEOs has been demonstrated across multiple oxide frameworks, encompassing rock salt, spinel, perovskite, PBAs, and layered structures. These materials exhibit superior electrochemical properties attributable to the unique cation configurations, with performance metrics (including specific capacity, cycle life, and rate capability) being precisely tunable through strategic cation substitution. Such attributes position HEOs as highly promising candidates for next-generation electrode and electrolyte materials in advanced battery systems.

Building on these fundamental insights, recent advancements have successfully enabled the development of high-performance, cobalt-free high-entropy battery electrode materials. These innovations have not only significantly enhanced battery safety but also reduced manufacturing costs. In LIBs, the pronounced lattice distortion effect inherent in HEAs effectively suppresses cation short-range ordering, thereby facilitating rapid Li^+^ diffusion and enhancing electrochemical performance. Furthermore, structural stability and conductivity have been substantially improved through advanced material engineering strategies, including surface coating and controlled nanopore formation. The unique architecture of HEMs demonstrates a remarkable ability to mitigate stress induced by Li^+^ intercalation, effectively preventing structural collapse caused by volume expansion while maintaining stability against rapid Li^+^ shuttling effects. Particularly in high-entropy layered oxides, the presence of two-dimensional ion migration channels between layers enables highly efficient Li^+^ transport. However, operational challenges persist, as the formation of oxygen vacancies and reduced metal species on material surfaces during cycling can impede Li⁺ intercalation, leading to accelerated capacity fading. To address this limitation, innovative approaches involving the partial substitution of oxygen anions with fluoride anions have been implemented, resulting in simultaneous improvements in both discharge-specific capacity and cycling stability. Collectively, HEMs exhibit exceptional structural integrity, superior ionic conductivity, and large dielectric constants, making them especially effective in addressing critical battery challenges such as electrolyte precipitation and low-temperature crystallization. These comprehensive improvements position HEMs as highly promising candidates for next-generation energy storage systems.

Leveraging the unique advantages of HEMs, recent breakthroughs in HEMs have led to the development of both liquid and solid-state systems exhibiting exceptional ionic conductivity and outstanding performance under extreme operational conditions, including high pressure and ultra-low temperatures. For instance, in LSBs, HEMOs with uniformly distributed multi-metal active sites effectively address the persistent challenges of polysulfide shuttle and sluggish redox kinetics. Particularly, HE MXenes, with the superior electrical conductivity and abundance of metal active sites, facilitate efficient chemical adsorption of LiPSs, thereby enhancing electrochemical performance. HEMs also exhibit exceptional catalytic activity and stability across a range of electrochemical environments, from alkaline to highly corrosive systems. In ZABs, these materials maintain high power density and record-breaking cycling stability. HEOs have emerged as highly versatile electrocatalysts, delivering outstanding performance in OER, ORR, and HER. In SIBs, the deliberate introduction of oxygen vacancies in high-entropy layered oxides significantly enhances Na^+^ transport kinetics, resulting in ultra-long cycle life and superior capacity retention. Phase engineering studies further demonstrate that precise tuning of O3-to-P2 phase ratios optimizes both capacity and structural stability across broad temperature ranges. For PIB systems, high-entropy metal disulfides exhibit dual functionality by simultaneously reducing K^+^ diffusion barriers and mitigating shuttle effects. Moreover, the integration of ML approaches has significantly accelerated the discovery and optimization of HEAs and high-entropy diborides. Through predictive modeling and high-throughput screening, these computational tools enable efficient exploration of the vast compositional space inherent to HEMs, facilitating the design of next-generation materials with tailored properties.

Despite the remarkable properties of HEMs—including exceptional structural stability, superior mechanical strength, outstanding corrosion resistance, and enhanced ion transport capabilities, which collectively enable extended operational lifetimes under extreme conditions, suppression of structural deformation, and improved electrochemical performance—significant challenges remain in translating these advances into commercially viable technologies. To address these barriers and accelerate the practical deployment of HEMs, a comprehensive development strategy centered on three critical dimensions is proposed: (1) preparation methods, (2) advanced materials characterization, and (3) advanced design. This multi-faceted approach aims to bridge the gap between laboratory-scale innovation and industrial-scale application, thereby unlocking the full potential of HEMs for next-generation energy storage and conversion technologies.

(1)**Preparation method:** Current synthesis of HEM synthesis predominantly depends on high-temperature processing to attain stable structures, however, achieving precise structural control under such conditions remains technically challenge. The industrial adoption of HEMs faces three primary obstacles: (1) prohibitively high synthesis costs arising from the use of precious metal constituents (e.g., Co/Ni-based HEAs costing), (2) energy-intensive high-temperature treatments (>1200 ℃), and (3) elemental segregation during processing. Emerging solutions are addressing these challenges through innovative approaches, particularly liquid-phase synthesis techniques such as electrochemical deposition and solvothermal methods. These approaches enable HEM fabrication below 400 ℃, substantially reducing energy consumption and material costs by facilitating precursor recycling. Additionally, AM technologies offer unprecedented precision in synthesis control while facilitating scalable production. For practical commercialization, prioritizing cost-effective mass production methods, particularly continuous flow liquid-phase systems, will be essential to transition HEMs from laboratory-scale curiosities to industrial-scale energy materials.

(2)**Advanced materials characterization:** The electrochemical performance of HEBMs cannot be simply attributed to multi-element synergistic effects, but requires in-depth investigation of the intrinsic atomic-scale mechanisms. Key scientific questions include: (1) the dynamic phase transition behavior between O3, P2, and P3 phases during electrochemical cycling, and (2) the interaction mechanisms among multiple components during charge/discharge processes. To address these challenges, advanced in situ characterization techniques are indispensable. Synchrotron radiation X-ray diffraction enables real-time tracking of crystal structure evolution with millisecond resolution; neutron diffraction accurately resolves the distribution characteristics of light elements such as Li/Na; and in-situ X-ray absorption spectroscopy reveals electronic structure evolution during charge compensation. By integrating multi-scale characterization approaches, including: pair distribution function analysis for local atomic environments; X-ray absorption near-edge structure for monitoring valence state changes, and high-resolution transmission electron microscopy for morphological evolution observation, precise structure–property relationships can be established. These insights offer critical theoretical guidance for the rational design of next-generation HEBM electrodes with optimized phase stability, ion transport pathways, and redox activity distribution. Such fundamental understanding will drive the transition of HEMs from empirical exploration toward predictive, design-driven development.

(3)**Advanced design:** The discovery and optimization of HEMs pose unique challenges that go beyond conventional trial-and-error approaches, necessitating the integration of advanced data-driven methodologies. ML techniques, particularly ANNs, have emerged as powerful tools to predict and screen novel HEAs by establishing quantitative structure–property relationships based on extensive materials datasets. Effective ML models for HEMs must incorporate critical thermodynamic parameters (e.g., configurational entropy *ΔS*_*conf*_ and *ΔH*_*mix*_ for phase stability assessment) and microstructural descriptors (including atomic size difference *δ* and valence electron concentration *VEC*), while experimental validation remains essential to verify computational predictions and address synthesis–structure–property discrepancies. However, current limitations include data scarcity, inconsistencies in experimental protocols across research groups, and computational costs associated with first-principles calculations. To advance the field, future efforts should focus on establishing standardized experimental protocols, developing comprehensive open-access material databases with detailed metadata, and implementing active learning frameworks that enable iterative model refinement through experimental feedback, thereby transforming ML from a predictive tool into a robust platform for accelerated HEMs discovery and development.
